# 40 Hz light flickering facilitates the glymphatic flow via adenosine signaling in mice

**DOI:** 10.1038/s41421-024-00701-z

**Published:** 2024-08-06

**Authors:** Xiaoting Sun, Liliana Dias, Chenlei Peng, Ziyi Zhang, Haoting Ge, Zejun Wang, Jiayi Jin, Manli Jia, Tao Xu, Wei Guo, Wu Zheng, Yan He, Youru Wu, Xiaohong Cai, Paula Agostinho, Jia Qu, Rodrigo A. Cunha, Xuzhao Zhou, Ruiliang Bai, Jiang-fan Chen

**Affiliations:** 1 The Eye and Brain Center, State Key Laboratory of Ophthalmology, Optometry and Visual Science, Eye Hospital, Wenzhou Medical University, Wenzhou, Zhejiang China; 2 Oujiang Laboratory (Zhejiang Laboratory for Regenerative Medicine, Vision and Brain Health), School of Ophthalmology & Optometry and Eye Hospital, Wenzhou Medical University, Wenzhou, Zhejiang China; 3 CNC-Center for Neurosciences and Cell Biology, University of Coimbra, Coimbra, Portugal; 4 FMUC-Faculty of Medicine, University of Coimbra, Coimbra, Portugal; 5 Department of Pediatric Sleep, The Second Affiliated Hospital and Yuying Children’s Hospital of Wenzhou Medical University, Wenzhou, Zhejiang China; 6 Key Laboratory of Biomedical Engineering of Education Ministry, College of Biomedical Engineering and Instrument Science, Zhejiang University, Hangzhou, Zhejiang China; 7 Interdisciplinary Institute of Neuroscience and Technology, Zhejiang University School of Medicine, Hangzhou, Zhejiang China; 8 Liangzhu Laboratory, MOE Frontier Science Center for Brain Science and Brain-machine Integration, State Key Laboratory of Brain-machine Intelligence, Zhejiang University, Hangzhou, Zhejiang China; 9 NHC and CAMS Key Laboratory of Medical Neurobiology, Zhejiang University, Hangzhou, Zhejiang China

**Keywords:** Molecular biology, Cell biology

## Abstract

The glymphatic-lymphatic system is increasingly recognized as fundamental for the homeostasis of the brain milieu since it defines cerebral spinal fluid flow in the brain parenchyma and eliminates metabolic waste. Animal and human studies have uncovered several important physiological factors regulating the glymphatic system including sleep, aquaporin-4, and hemodynamic factors. Yet, our understanding of the modulation of the glymphatic system is limited, which has hindered the development of glymphatic-based treatment for aging and neurodegenerative disorders. Here, we present the evidence from fluorescence tracing, two-photon recording, and dynamic contrast-enhanced magnetic resonance imaging analyses that 40 Hz light flickering enhanced glymphatic influx and efflux independently of anesthesia and sleep, an effect attributed to increased astrocytic aquaporin-4 polarization and enhanced vasomotion. Adenosine-A_2A_ receptor (A_2A_R) signaling emerged as the neurochemical underpinning of 40 Hz flickering-induced enhancement of glymphatic flow, based on increased cerebrofluid adenosine levels, the abolishment of enhanced glymphatic flow by pharmacological or genetic inactivation of equilibrative nucleotide transporters-2 or of A_2A_R, and by the physical and functional A_2A_R–aquaporin-4 interaction in astrocytes. These findings establish 40 Hz light flickering as a novel non-invasive strategy of enhanced glymphatic flow, with translational potential to relieve brain disorders.

## Introduction

The glymphatic-lymphatic system is increasingly recognized as fundamental for the homeostasis of the brain milieu since it defines cerebral spinal fluid (CSF) flow in the brain parenchyma and eliminates metabolic waste^1^. The glymphatic system involves CSF flowing into brain parenchyma along the perivascular space by the pulsatile arterial activity^2^, CSF-interstitial fluid (ISF) exchange through the polarized distribution of the water-permeable channel aquaporin-4 (AQP4) in astrocytic endfeet^3,4^, followed by a clearance efflux of the CSF and extracellular waste through the lymphatic system^5^. The glymphatic clearance is mostly active during sleep^6^ and its disturbance or decreased function on aging^7^, leads to the accumulation of pathogenic proteins in the brain such as tau^8,9^, β-amyloid^10^, and α-synuclein^11^. The intertwined relationship of sleep, aging, glymphatic clearance, and accumulation of pathogenic proteins suggests that glymphatic failure is a critical step in the pathogenesis of a broad range of brain injuries/disorders and may represent a therapeutically targetable final common pathway. Recent studies have uncovered some physiological factors regulating the glymphatic system including sleep, AQP4, and hemodynamic factors. Yet, our understanding of the modulation of the glymphatic system is limited, which has hindered the development of glymphatic-based treatment for aging and neurodegenerative disorders.

High-frequency sensory stimulation is increasingly recognized as a promising non-invasive strategy to modulate brain function, affording benefits in various pathological conditions^12–14^. This is best heralded by the designation by the US FDA of combined visual and acoustic stimulation (GENUS) as a “breakthrough medical devise” for the treatment of Alzheimer’s disease (AD)^15^. In fact, GENUS reduces β-amyloid deposit and reverses cognitive deficits in AD mouse models^12,13^, reduces ventricular dilation and hippocampal atrophy, and improves memory performance in mild AD patients^15^. In addition, 40 Hz light flickering attenuates pathological features of ischemia^16^ and traumatic brain injury^17^, and promotes sleep^18^. 40 Hz light flickering can entrain gamma oscillations across multiple brain regions^13,15^, activates microglia^12^, influences vascular densities, and neuroimmune signaling^14^, bolsters levels of neuromodulators such as acetylcholine^19^, and alters gene expression profiles in many brain cell types^12^.

Two recent discoveries which we made on 40 Hz flickering effects raise the exciting possibility that the glymphatic-lymphatic system may represent a mechanism engaged by 40 Hz light flickering to control brain injury. First, brief 40 Hz light flickering produces a robust and sustained increase in the extracellular adenosine levels in the primary visual cortex and other brain regions^18^. Second, 40 Hz light flickering promotes sleep^18^, when the glymphatic system is mostly active^6^. Adenosine-A_2A_ receptor (A_2A_R) signaling is uniquely positioned to control the glymphatic system based on its role as homeostatic regulator of sleep “need”^20^ and its ability to regulate AQP4 polarization^21^ and cerebral blood flow^22^, both key factors supporting glymphatic flow^3,6,23^. This novel relation between 40 Hz flickering, enhanced adenosine signaling and sleep, which bolsters the glymphatic flow, prompted our proposal that 40 Hz light flickering represents a non-invasive approach to facilitate the glymphatic flow via adenosine signaling.

In this study, we discovered that brief 40 Hz light flickering robustly enhanced glymphatic influx and efflux in a frequency-dependent manner in mice, independently of anesthesia and sleep, as concluded using fluorescence tracing in ex vivo brain slices, two-photon microscopy in awake mice, and in vivo dynamic contrast-enhanced magnetic resonance imaging (DCE-MRI). This effect is attributed to increased AQP4 polarized expression in astrocyte endfeet and enhanced vasomotion. Furthermore, we identified adenosine-A_2A_R signaling as a molecular underpinning of 40 Hz flickering-induced enhancement of glymphatic flow, as concluded by the increased adenosine levels in the CSF, by the abolishment upon pharmacological and genetic inactivation of equilibrative nucleoside transporters (ENTs) of 40 Hz light flickering-induced increase in glymphatic flow and by the physical and functional interaction of A_2A_R and AQP4 in cultured astrocytes. Collectively, these findings established 40 Hz light flickering as a novel non-invasive strategy and adenosine-A_2A_R signaling as an important modulator to enhance glymphatic flow, with translational potential to therapeutically relieve neurological disorders.

## Results

### 40 Hz light flickering enhances glymphatic influx and efflux measured by CSF fluorescence tracing

**Fig. 1:**
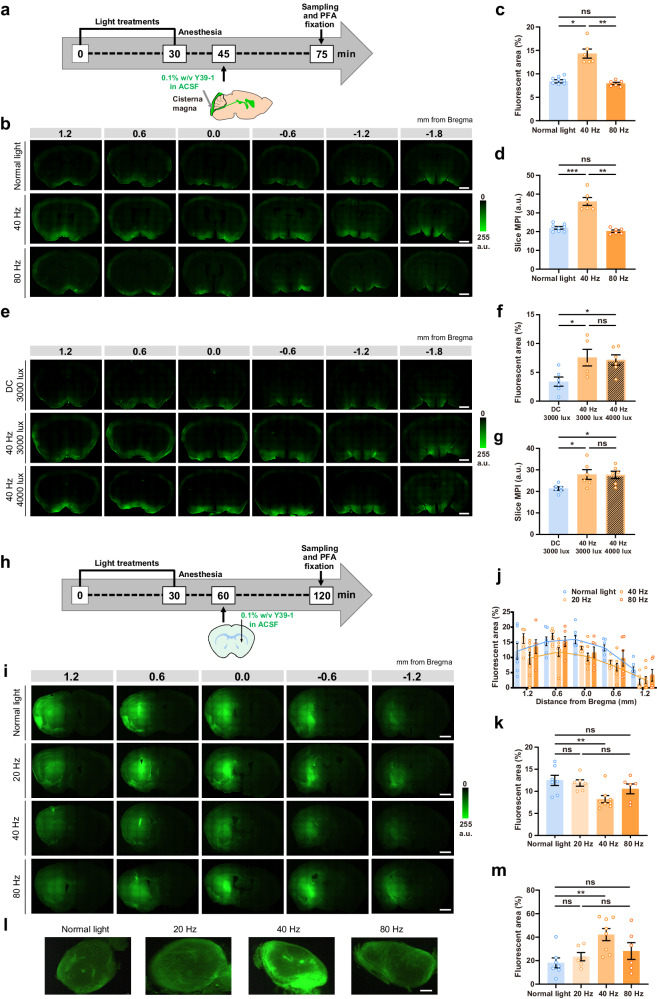
40 Hz light flickering enhances glymphatic influx and efflux measured by CSF fluorescence tracing. **a** Schematic protocol of the measurement of cerebrospinal fluid influx after exposure for 30 min to control light (500 lux) or light flickering (white light, illuminance of 3000 lux, irradiance of 1.10 mW/cm^2^ at 20 cm distance, 50% duty cycle) at frequencies of either 40 Hz or 80 Hz, followed by injecting the fluorescent CSF tracer (Y39-1) into the cisterna magna; after 30 min mice were sacrificed and Y39-1 fluorescence was measured in coronal brain sections. **b** Representative images showing that 40 Hz light flickering increased the parenchymal distribution of Y39-1 at 30 min after intracisternal injection; numbers indicate the anteroposterior distance from bregma in mm (scale bars, 1 mm). **c**, **d** Quantification of intracisternally injected Y39-1 mean pixel intensity (MPI, in arbitrary units, a.u.) and fluorescent area (expressed as % of section area) in whole sections 30 min after exposure to 40 Hz or 80 Hz light flickering during 30 min; analysis was performed on six sections per animal (*n* = 8 mice normal light, *n* = 6 mice 40 Hz, *n* = 6 mice 80 Hz, mean ± SEM in the bar graphs, ****P < 0.0001, ns, not significant, one-way ANOVA with Tukey’s multiple comparison test). **e** Representative images showing that 40 Hz light flickering at 3000 lux increased glymphatic influx, with similar effects at 4000 lux, compared to DC light stimulation at 3000 lux; numbers indicate the anteroposterior distance from bregma in mm (scale bars, 1 mm). **f**, **g** Quantification of intracisternally injected Y39-1 MPI (in arbitrary units, a.u.) and fluorescent area (expressed as % of section area) in whole sections; analysis was performed in six sections per animal (*n* = 6 mice/group, mean ± SEM in the bar graphs, *P < 0.05, ns, not significant, one-way ANOVA with Tukey’s multiple comparison test). **h** Schematic protocol of the measurement of CSF efflux after exposure for 30 min to control light or light flickering at frequencies of either 20, 40, or 80 Hz, followed by injecting the fluorescent CSF tracer (Y39-1) intrastriatally; after 60 min mice were sacrificed and Y39-1 fluorescence was measured in coronal brain sections. **i** Representative images showing that 40 Hz light flickering selectively decreased the Y39-1 fluorescence in the brain parenchyma; numbers indicate the anteroposterior distance from bregma in mm (scale bars, 1 mm). Quantification of the area (expressed as % of section area) covered by Y39-1 in coronal brain sections (**j**) and mean fluorescence (**k**) after exposure to 20, 40 or 80 Hz light flickering; analysis was performed in five sections per animal (*n* = 6–8 mice/group, mean ± SEM in the bar graphs, **P < 0.01, ns, not significant, one-way ANOVA with Tukey’s multiple comparison test). **l** Representative images showing that 40 Hz light flickering selectively increased the Y39-1 fluorescence in the deep cervical lymph nodes (scale bar, 100 μm). **m** Quantification of the area (expressed as % of section area) covered by Y39-1 in the deep cervical lymph nodes after exposure to 20, 40 or 80 Hz light flickering (mean ± SEM in the bar graphs, **P < 0.01, ns, not significant, one-way ANOVA with Tukey’s multiple comparison test).

We first evaluated the frequency-dependent effects of light flickering on glymphatic influx in wild-type (WT) mice, by injecting a CSF fluorescent tracer (Y39-1, 2 kDa) into the subarachnoid CSF of the cisterna magna, then following its distribution in the brain parenchyma over time (Fig. 1a). Previous studies have established that the percentage of fluorescent area and the mean fluorescence intensity quantitatively reflect the glymphatic flow^24,25^. We observed that a 30-min exposure to light flickering (white light, illuminance of 3000 lux, irradiance of 1.10 mW/cm^2^ at 20 cm distance, 50% duty cycle) at 40 Hz, but not at 80 Hz, significantly increased within 30 min the parenchymal distribution of the CSF tracer compared to normal room illumination (500 lux) (Fig. 1b–d). To exclude the possibility of unexpected molecular processes affecting the distribution of tracer Y39-1, as the α-synuclein Y39-1 peptide has a core amino acid sequence that forms β-helix sheets contributing its aggregation^26^, we designed and generated a Y39-1-mut peptide, which has a similar molecular size but has a denatured structure compared to Y39-1. The results show that both peptides yield similar distribution patterns (Supplementary Fig. S1), indicating that fluid dynamics (rather than amino acid sequence) are the determinants of the tracer’s distribution. Furthermore, we also tested the effect of 20 Hz light flickering to demonstrate the frequency-dependent effects of light flickering on glymphatic activity. As our previous data showed that 20 and 80 Hz are not capable of producing gamma oscillation in V1 or generating biological effects like sleep^18^, we chose 20 Hz as a representative low-frequency band in this study. Additionally, we conducted an additional control experiment wherein we compared glymphatic influx in mice subjected to 40 Hz light flickering vs direct current (DC) light stimulation at 3000 lux (Fig. 1e–g). The findings further validate that 40 Hz light flickering at 3000 lux increased glymphatic influx compared to DC at 3000 lux. Moreover, our recent report found that 40 Hz flickering under high light intensities (3000 lux, 4000 lux, and 6000 lux) significantly and persistently increased extracellular adenosine^18^. However, 40 Hz light flickering at 3000 lux and 4000 lux produced similar effects on glymphatic influx (Fig. 1e–g), so we chose 3000 lux to explore the impact of light flickering on glymphatic flow at a molecular level.

We next evaluated the impact of 40 Hz light flickering on CSF efflux out of the brain parenchyma, one hour after an interstitial Y39-1 injection (Fig. 1h). As shown in Fig. 1i–m, mice exposed to 40 Hz (but not 20 or 80 Hz) light flickering displayed a lower fluorescence within the striatum (Fig. 1i–k) and a higher fluorescence in the deep cervical lymph nodes (dcLNs; Fig. 1l, m), indicating an increased glymphatic efflux. Taken together, a brief 30-min 40 Hz light flickering can efficaciously enhance glymphatic flow, as heralded by an increased influx and efflux of CSF fluorescence tracers.

### 40 Hz flickering increases glymphatic influx independently of sleep and anesthesia

**Fig. 2:**
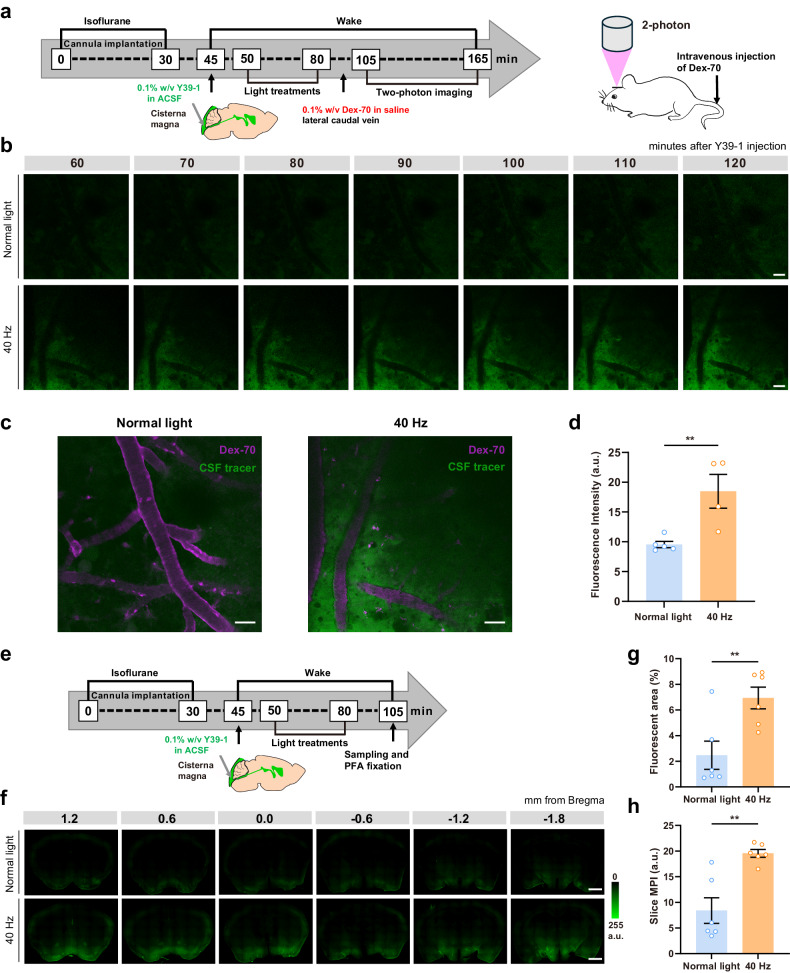
40 Hz light flickering enhances glymphatic influx independently of sleep and anesthesia. **a** Schematic protocol of in vivo two-photon microscopy to directly observe the impact of 40 Hz flickering on glymphatic flow in awake mice. **b** Representative in vivo two-photon images (100 μm below the cortical surface) repeatedly scanned at 10-min intervals showing that after 30 min of exposure to 40 Hz light flickering, the parenchymal distribution of Y39-1 (following its intracisternal injection) was significantly enhanced compared to mice exposed to normal light (scale bars, 50 μm). **c** Representative images (100 μm below the cortical surface, vasculature in magenta and Texas Red-conjugated dextran-70 kDa administered via the caudal vein) at 2 h after intracisternal injection showing that 40 Hz light flickering increased CSF tracer influx (scale bars, 50 μm). **d** Quantitative analysis of intracisternally injected Y39-1 fluorescence intensity in images 80–100 μm below the cortical surface (arbitrary units, a.u.) at 2 h after intracisternal injection (*n* = 4–5 mice/group, mean ± SEM in the bar graphs, **P < 0.01, unpaired Student’s *t*-test). **e** Schematic protocol of pre-implanted injection of a guide cannula into the cisterna magna before administering the glymphatic tracer Y39-1. **f** Representative images showing that 40 Hz light flickering increased glymphatic influx in awake mice with pre-implanted cannulas (scale bars, 1 mm). **g**, **h** Quantification of intracisternally injected Y39-1 MPI (in arbitrary units, a.u.) and fluorescent area (expressed as % of section area) in whole sections 30 min after exposure to 40 Hz light flickering or normal light during 30 min (*n* = 6 mice/group, mean ± SEM in the bar graphs, ** *P* < 0.01, unpaired Student’s *t*-test).

The regulation of glymphatic flow is robustly influenced by sleep–wake stages and exhibits a strong association with slow-wave activity, specifically with delta power^27^. To test whether 40 Hz flickering can modulate glymphatic activity through both sleep-dependent as well as sleep-independent mechanisms, we employed two-photon microscopy to directly and continuously monitor the impact of 40 Hz flickering on glymphatic flow in awake mice (Fig. 2a). We report that these awake mice exposed to 40 Hz light flickering for 30 min, displayed an increased parenchymal distribution of CSF tracer compared to mice exposed to normal light (Fig. 2b); quantitative analysis of fluorescence intensity shows that 40 Hz light flickering increased CSF tracer influx at 2 h after its intracisternal injection in these awake mice (Fig. 2c, d). In parallel, we conducted Electroencephalography (EEG) recordings on mice exposed to the same environmental conditions and treatments as those used in the two-photon experiments, to confirm that the mice were indeed in an awake state during the two-photon experiments (Supplementary Fig. S2). These findings reinforce our conclusion that 40 Hz flickering improves glymphatic influx in awake animals, on top of and/or independently of the effect of 40 Hz on sleep then impacting glymphatic flow.

The two-photon experiment also demonstrated that 40 Hz flickering improves glymphatic influx independently of anesthesia, which is another important factor affecting glymphatic activity^6^. Additionally, we conducted ex vivo experiments, in which guide cannulas were pre-implanted into the cisterna magna prior to administering the glymphatic tracer (Fig. 2e). This procedure allows us to avoid the possible confounding effect on the glymphatic activity of the anesthesia used to implant the guide cannula shortly before measuring glymphatic activity. We confirmed that 40 Hz light flickering still increased glymphatic influx in mice with pre-implanted cannulas (Fig. 2f–h), which confirms that this effect is independent of anesthesia. Collectively, these in vivo and ex vivo experiments unequivocally demonstrated that 40 Hz flickering can increase glymphatic influx independently of anesthesia and sleep.

### 40 Hz light flickering induces a global increase in glymphatic influx measured by DCE-MRI

**Fig. 3:**
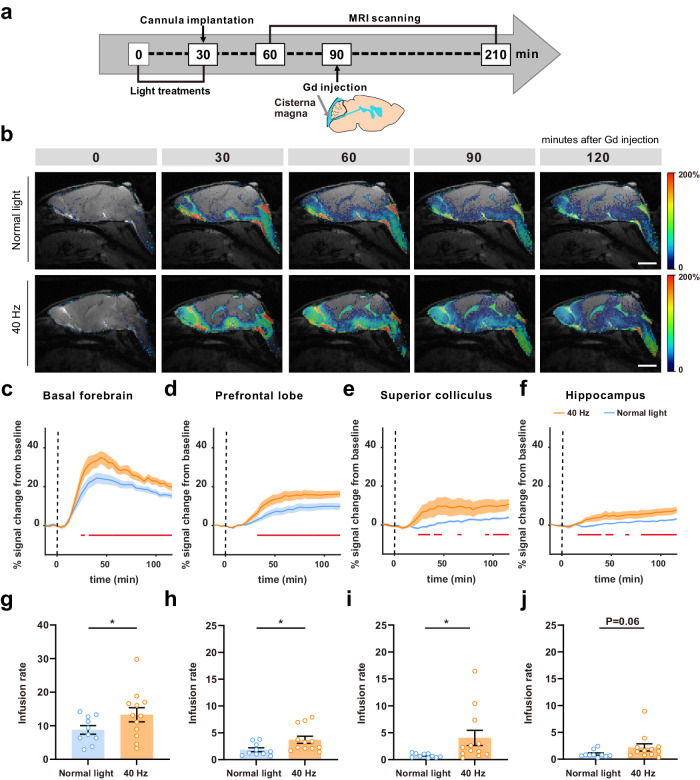
40 Hz light flickering increases glymphatic flow measured by DCE-MRI. **a** Schematic protocol of DCE-MRI. **b** Representative pseudocolour-scaled sagittal images of brains from mice exposed to normal light or 40 Hz light flickering; numbers indicate the time elapsed after injecting the Gd-DTPA tracer into the cisterna magna. (scale bars, 3 mm). **c**–**f** Signal intensity plots based on % signal change from baseline in time signal curves, triggered by either normal light or 40 Hz light flickering and acquired from the basal forebrain, prefrontal lobe, hippocampus and superior colliculus, demonstrated an earlier rise in signal intensity following Gd-DTPA injection and a greater overall elevation (the horizontal red lines represent time points with statistically significant differences). **g**–**j** Quantification of the infusion rate demonstrated a faster increase in signal prior to reaching its peak after exposure to 40 Hz light flickering (each dot represents a region of the hemisphere, *n* = 5 mice exposed to normal light, *n* = 6 mice exposed to 40 Hz, mean ± SEM in the bar graphs, *P < 0.05, unpaired Student’s *t*-test).

We further resorted to DCE-MRI to dynamically visualize CSF tracer distribution over time in mice exposed to normal light or 40 Hz light flickering. A magnetic resonance contrast agent (Gd-DTPA, 938 Da) was infused via the cisterna magna and a T1-weighted FLASH sequence in the sagittal plane was acquired continuously for 2 h (Fig. 3a). Mice exposed to 40 Hz light-flickering exhibited an earlier rise in signal intensity following Gd-DTPA injection and a greater overall signal elevation in multiple brain regions (Fig. 3b; Supplementary Video S1), namely in the basal forebrain, prefrontal lobe, superior colliculus and hippocampus (the horizontal red lines represent time points with statistically significant differences, Fig. 3c–f). Additionally, there was a faster infusion rate in the basal forebrain, prefrontal lobe and superior colliculus, with a trend observed in the hippocampus after exposure to 40 Hz light flickering (Fig. 3g–j). These results suggest an enhanced influx and improved flow of the CSF tracer via the glymphatic system in the brain parenchyma.

### The enhancement of the glymphatic influx by 40 Hz light flickering involves astrocytic AQP4 polarization

**Fig. 4:**
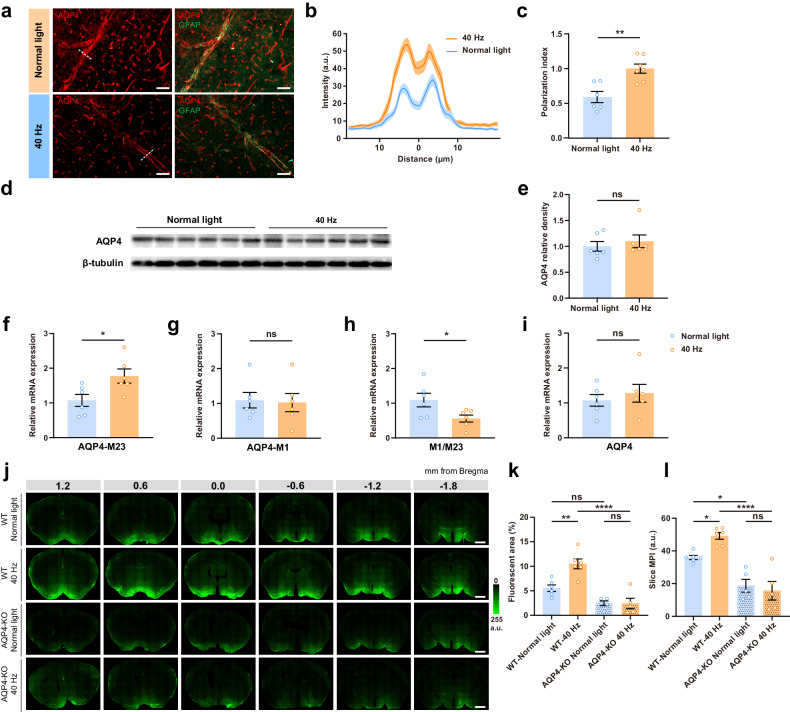
40 Hz light flickering enhances the glymphatic influx through astrocytic AQP4 polarization. **a** Immunotstaining images of AQP4 (red) and of the astrocytic marker glial fibrillary acidic protein (GFAP, green) in brain sections fixed 60 min after exposure to either normal light or 40 Hz light flickering for 30 min (scale bars, 50 μm). **b** Average intensity of AQP4 staining centered on the vasculature in the cerebral cortex after exposure to either normal light or 40 Hz light flickering, with mean ± SEM indicated by the thick line (mean) with shading (SEM). **c** The average AQP4 polarization index increased after 40 Hz light flickering; the quantification of AQP4 polarization was carried out upon setting the baseline as the average intensity within a 10 μm range, from –20 μm to –10 μm relative to the point of peak fluorescence (*n* = 6 mice normal light, *n* = 7 mice 40 Hz, mean ± SEM in the bar graph, **P < 0.01, unpaired Student’s *t*-test). **d** Representative western blot image (out of 3 similar experiments) and quantification. **e** AQP4 and β-tubulin densities in whole brain extracts after exposure to either normal light or 40 Hz light flickering (*n* = 6 mice/group, mean ± SEM in bar graphs, ns, not significant, unpaired Student’s *t*-test). **f**–**i** Effect of 40 Hz light flickering on mRNA expression of AQP4-M23 (**f**), AQP4-M1 (**g**), on the ratio of M1/M23 (**h**) and AQP4 (**i**), assessed by reverse transcription PCR (RT-qPCR) in whole brain extracts (*n* = 6 mice/group, mean ± SEM in the bar graphs, *P < 0.05, ns, not significant, unpaired Student’s *t*-test). **j** Representative photographs of fluorescence in coronal brain sections collected 30 min after injection of the Y39-1 fluorescent tracer in the cisterna magna of WT mice (top two rows) or *AQP4*-KO mice (bottom two rows), previously exposed either to normal light or 40 Hz light flickering for 30 min; numbers indicate the anteroposterior distance from bregma in mm (scale bars, 1 mm). **k**, **l** Quantification of intracisternally injected Y39-1 MPI (in arbitrary units, a.u.) and of the fluorescent area (expressed as % of section area), showed that exposure to 40 Hz light flickering increased glymphatic influx in WT mice but not in *AQP4*-KO mice; analysis was performed on six sections per animal (*n* = 5–6 mice/group, mean ± SEM in the bar graphs, *P < 0.05, **P < 0.01, ****P < 0.0001, ns, not significant, one-way ANOVA with Tukey’s multiple comparison test).

The polarization of AQP4, a water channel present at the endfeet of astrocytes forming the outer barrier of the perivascular spaces, is critical for the exchange between the paravascular CSF and ISF^3,4^, and is a main driver of the glymphatic system. We now report that the perivascular AQP4 polarization on cortical astrocytes significantly increased 30 min after 40 Hz light flickering (Fig. 4a–c), without significant differences in total brain AQP4 density between normal light and 40 Hz light flickered mice (Fig. 4d, e). The localization of AQP4 can be influenced by splice variants, specifically M1 or M23, the latter being more abundant at astrocytic endfeet^28,29^. Accordingly, we found that 40 Hz light flickering increased the gene expression of AQP4-M23 (Fig. 4f) and downregulated AQP4-M1/M23 ratio (Fig. 4h) without altering AQP4-M1 (Fig. 4g) and total AQP4 (Fig. 4i). Overall, these findings prompt the hypothesis that 40 Hz light flickering increased glymphatic influx through and increased astrocytic AQP4 polarization.

This hypothesis was further experimentally tested by evaluating paravascular CSF influx in *AQP4*-knockout (*AQP4*-KO) mice. We first confirmed that *AQP4*-KO mice displayed a significantly diminished influx of the CSF tracer when compared to their WT littermates, consistent with previous reports^1,3^. Moreover, 40 Hz light flickering failed to alter CSF influx in *AQP4*-KO mice (Fig. 4j–l). These findings indicate that the 40 Hz light flickering-induced enhancement of the glymphatic flow is strictly dependent on AQP4.

### 40 Hz light flickering increases vasomotion and cerebral blood flow (CBF)

**Fig. 5:**
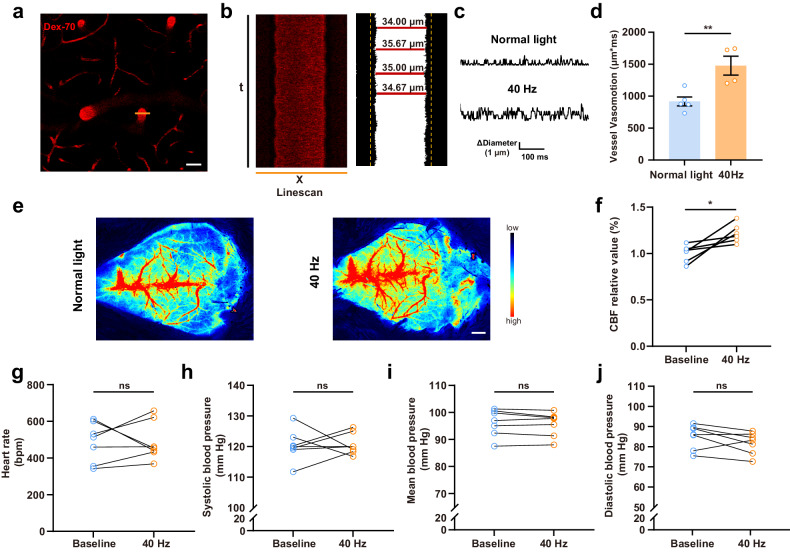
40 Hz light flickering enhances the glymphatic influx through increased arterial vasomotion. **a** High-frequency orthogonal line scans were generated across surface and penetrating arteries (scale bar, 50 μm). **b** Representative raw X–t scans were processed with thresholding to enhance edge detection, allowing for the measurement of luminal diameter over time. **c** Representative images of vasomotion after 40 Hz light flickering or exposure to normal light in awake mice. **d** Vasomotion was measured from ascending arteries: after exposure to 40 Hz light flickering for 30 min, arterial vasomotion was significantly higher than after exposure to normal light in these awake mice. **e**, **f** Representative images (scale bar, 1 mm) of laser speckle flowmetry showing that 40 Hz light flickering increased cortical cerebral blood flow in WT mice, as quantified in **f** (*n* = 6 mice/group, mean ± SEM in the bar graphs, *P < 0.05, paired Student’s *t*-test). **g**–**j** 40 Hz light flickering did not significantly modify either heart rate, systolic blood pressure, mean blood pressure or diastolic blood pressure, measured using a non-invasive tail-cuff method (*n* = 7 mice/group, mean ± SEM in the bar graphs, ns, not significant, paired Student’s *t*-test).

Since vasomotion is a driving force for CSF into the brain parenchyma along paravascular pathways^30^, we next evaluated the impact of 40 Hz light flickering on vasomotion using two-photon microscopy in awake mice injected with Texas Red-conjugated dextran (70 kDa) via the caudal vein to visualize the vasculature through a cranial window. Changes in arterial diameter were measured by high-frequency line scanning. As previously described^7^, a value for “vasomotion” was extracted from X–t (diameter–time) plots (Fig. 5a, b), which integrated periods of 3 s. We observed a notable increase in arterial vasomotion in these awake mice after exposure to 40 Hz light flickering for 30 min (Fig. 5c, d).

It is well established that the glymphatic influx exhibits a correlation with CBF, which is a motor of glymphatic flow^20,31,32^. Accordingly, laser speckle flowmetry showed a significant increase in cortical CBF after 40 Hz light flickering in WT mice (Fig. 5e, f). Since the glymphatic flow also depends on cardiovascular and hemodynamic factors^2,23,33^, we tested the effect of 40 Hz light flickering on blood pressure and heart rate, measured using a non-invasive tail-cuff method with a BP-2010A blood pressure device. Hemodynamic monitoring revealed that there were no statistically significant differences in blood pressure or heart rate before and after 40 Hz light flickering in WT mice (Fig. 5g–j). Therefore, 40 Hz light flickering appears to enhance glymphatic flow by acting through vasomotion and CBF, without affecting blood pressure or heart rate.

### Adenosine released through ENT2 mediates the enhancement of glymphatic flow induced by 40 Hz light flickering

**Fig. 6:**
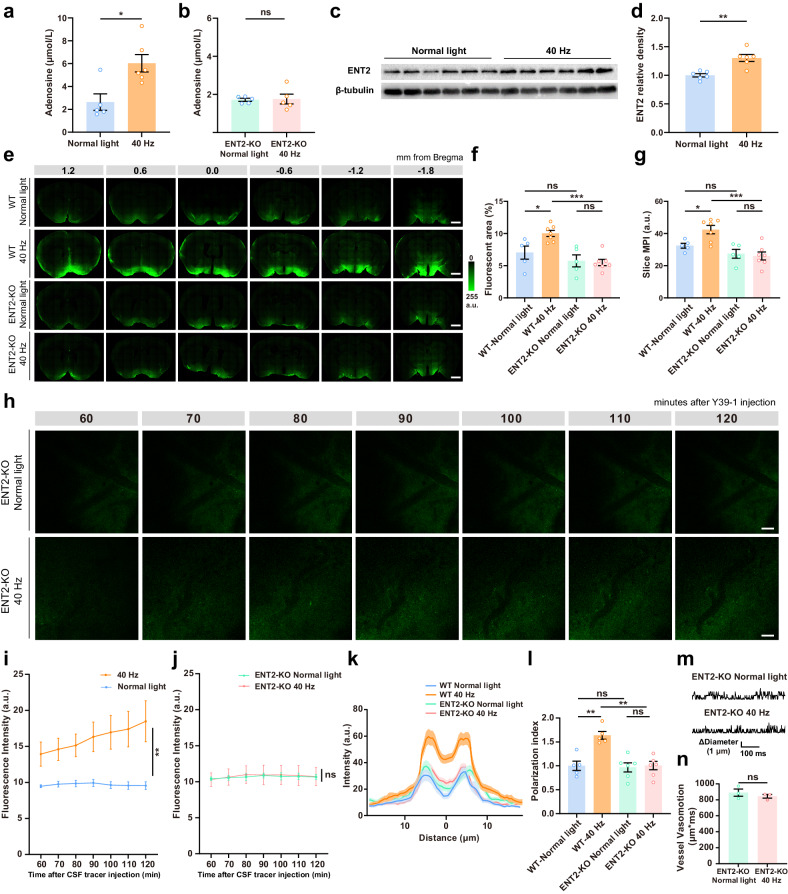
ENT2-mediated adenosine release mediates the enhancement of glymphatic flow induced by 40 Hz light flickering. **a**, **b** Levels of extracellular adenosine quantified by UPLC in the CSF collected from the cisterna magna of anesthetized WT mice (**a**) or *ENT2*-KO mice (**b**) 30 min after exposure either to control light or 40 Hz light flickering for 30 min (*n* = 5–7 mice/group, mean ± SEM in the bar graphs, *P < 0.05, ns, not significant, unpaired Student’s *t*-test). **c**, **d** Representative western blot (**c**, out of 3 similar experiments) and quantification (**d**) of ENT2 and β-tubulin densities 30 min after exposure to normal light or 40 Hz light flickering during 30 min (*n* = 6 mice/group, mean ± SEM in the bar graphs, **P < 0.01, unpaired Student’s *t*-test). **e** Representa*t*ive photographs of fluorescence in coronal brain sections collected 30 min after injection of the Y39-1 fluorescent tracer in the cisterna magna of WT mice (top two rows) or *ENT2*-KO mice (bottom two rows), previously exposed either to normal light or 40 Hz light flickering during 30 min; numbers indicate the anteroposterior distance from bregma in mm (scale bars, 1 mm). **f**, **g** Quantification of intracisternally injected Y39-1 MPI (in arbitrary units, a.u.) and of the fluorescent area (expressed as % of section area), showed that exposure to 40 Hz light flickering increased the glymphatic influx in WT mice, but not in *ENT2*-KO mice; analysis was performed in six sections per animal (*n* = 5–7 mice/group, mea*n* ± SEM in the bar graphs, *P < 0.05, ***P < 0.001, ns, not significant, one-way ANOVA with Tukey’s multiple comparison test). **h** Representative in vivo two-photon images (100 μm below the cortical surface) repeatedly scanned at 10-min intervals showing that after 30 min of exposure to 40 Hz light flickering, the parenchymal distribution of Y39-1 (following its intracisternal injection) was not significantly different compared to *ENT2*-KO mice exposed to normal light (scale bars, 50 μm). **i**, **j** Quantification of intracisternally injected Y39-1 fluorescence intensity (arbitrary units, a.u.) in images 80–100 μm below the cortical surface showing that *ENT2*-KO mice did not display an increased parenchymal distribution triggered by 40 Hz light flickering (*n* = 3–4 mice/group, **P < 0.01, ns, not significant, two-way repeated measures ANOVA). **k** Average intensity of AQP4 staining centered on the vasculature in the cerebral cortex after exposure to either normal light or 40 Hz light flickering in *ENT2*-KO mice, with mean ± SEM indicated by the thick line (mean) with shading (SEM). **l** The average AQP4 polarization index increased after 40 Hz light flickering; the quantification of AQP4 polarization was carried out upon setting the baseline as the average intensity within a 10 μm range, from –20 μm to –10 μm relative to the point of peak fluorescence (*n* = 5–6 mice/group, mean ± SEM in the bar graph, **P < 0.01, ns, not significant, one-way ANOVA with Tukey’s multiple comparison test). **m** Representative images of vasomotion after 40 Hz light flickering in *ENT2*-KO mice. **n** Vasomotion was measured from ascending arteries and the increased vasomotion after 40 Hz light flickering observed in WT mice was abolished in *ENT2*-KO mice (*n* = 5–7 mice/group, mean ± SEM in the bar graphs, ns, not significa*n*t, one-way ANOVA with Tukey’s multiple comparison test).

We next attempted to tackle the mechanisms underlying the impact of 40 Hz light flickering on the glymphatic system. Since adenosine is released in an activity-dependent manner^34^ and modulates CBF by acting at A_2A_R^22^, we hypothesized that adenosine could mediate the bolstering effect of 40 Hz light flickering on the glymphatic system. Accordingly, ultra-high performance liquid chromatography (UPLC) analysis revealed a significant increase in extracellular adenosine levels in the mouse CSF 30 min after the cessation of 40 Hz light flickering (Fig. 6a). Transmembrane adenosine transport is mediated by two distinct ENTs, namely the higher affinity but lower transporting capacity ENT1 and the lower affinity but higher transport capacity ENT2^35^. We confirmed that 40 Hz flickering-induced adenosine was abolished in *ENT2*-KO mice (Fig. 6b).

Furthermore, we found that transgenic mice with a deletion of ENT2^36^ did not display an increased CSF influx triggered by 40 Hz light flickering (Fig. 6e–g), which was preserved in *ENT1*-KO mice (Supplementary Fig. S3). Accordingly, 40 Hz light flickering significantly increased ENT2 density in whole-brain extracts (Fig. 6c, d). We further conducted two-photon microscopy to observe the impact of 40 Hz flickering on glymphatic flow in awake *ENT2*-KO mice. We found that *ENT2*-KO mice did not display an increased parenchymal distribution triggered by 40 Hz light flickering (Fig. 6h–j). After exposure to 40 Hz light flickering for 30 min, the parenchymal distribution of CSF tracer was not significantly different compared to *ENT2*-KO mice exposed to normal light (Fig. 6j). These results imply that ENT2 (but not ENT1) is the molecular pathway mediating the enhancement of extracellular adenosine responsible for the increase of glymphatic flow triggered by 40 Hz light flickering, which is consistent with our recent report^18^.

Lastly, we resorted to dipyridamole (an inhibitor for both ENT and phosphodiesterase, with IC_50_ values at 50 nM^37^ and 7 µM^38^, respectively, indicating a better affinity for ENT), to block adenosine outflow (Supplementary Fig. S4a). We found that administration of dipyridamole before (Supplementary Fig. S4b–d) and near the end of light stimuli (Supplementary Fig. S4e–g) abolished the increased glymphatic influx caused by 40 Hz light flickering. This suggests that adenosine released via ENT is a key mediator of the 40 Hz flickering-induced enhancement of the glymphatic flow. Besides, adenosine produced during the 30-minute light treatment period is not enough to increase the glymphatic influx indicating the effect brought by 40 Hz light flickering is a long-lasting hysteresis rather than an immediate reaction to the light stimuli.

There was no significant difference in the baseline levels of AQP4 in the *ENT2*-KO compared to WT littermates, consistent with the report by Chiang et al. (Supplementary Fig. S4h, i)^39^. However, we observed that the increased AQP4 polarization (Fig. 6k, l) and arterial vasomotion after 40 Hz light flickering were abolished in *ENT2*-KO mice (Fig. 6m, n). These findings demonstrate that 40 Hz light flickering facilitated glymphatic influx through regulation of AQP4 polarization and arterial vasomotion dependent on the engagement of increased ENT2 activity by 40 Hz light flickering.

### Adenosine-A_2A_Rs mediate the 40 Hz light flickering-induced increase in glymphatic flow

**Fig. 7:**
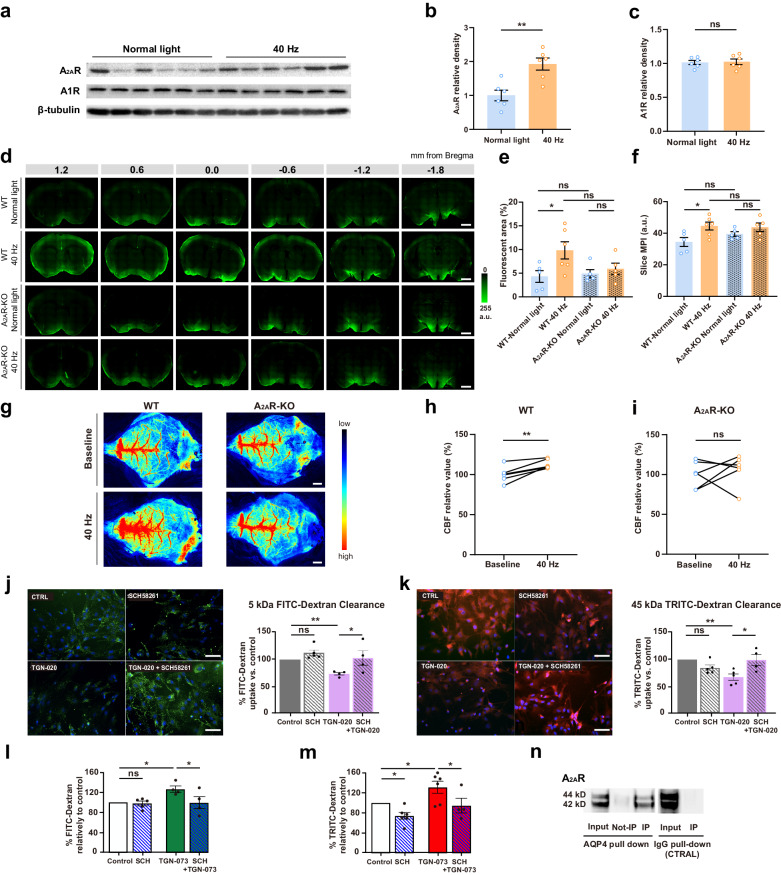
Adenosine-A_2A_Rs mediate the 40 Hz light flickering-induced increase of the glymphatic flow. **a**–**c** Representative western blot images (**a**, out of 3 similar experiments) and quantification (**b**, **c**) of A_2A_R, A_1_R, and β-tubulin in whole brain extracts after exposure to normal light or 40 Hz light flickering (*n* = 6 mice/group, mean ± SEM in the bar graphs, **P < 0.01, ns, not significant, unpaired Student’s *t*-test). **d** Representative photographs of fluorescence in coronal brain sections collected 30 min after injection of the Y39-1 fluorescent tracer in the cisterna magna of WT mice (top two rows) or *A*_*2A*_*R*-KO mice (bottom two rows), previously exposed either to normal light or to a 30 min period of 40 Hz light flickering; numbers indicate the anteroposterior distance from bregma in mm (scale bars, 1 mm). **e**, **f** Quantification of intracisternally injected Y39-1 MPI (in arbitrary units, a.u.) and of the fluorescent area (expressed as % of section area), showed that exposure to 40 Hz light flickering increased the glymphatic influx in WT mice, but not in *A*_*2A*_*R*-KO mice; analysis was performed on six sections per animal (*n* = 5–6 mice/group, mean ± SEM in the bar graphs, *P < 0.05, ns, not significant, one-way ANOVA with Tukey’s multiple comparison test). **g** Representative images of laser speckle flowmetry showing that 40 Hz light flickering increased cortical cerebral blood flow in WT mice, but not in *A*_*2A*_*R*-KO mice, as quantified in **h**, **i** (*n* = 6 mice/group, mea*n* ± SEM in the bar graphs, **P < 0.01, ns, not significant, paired Student’s *t*-test). **j**, **k** Representative pho*t*ographs (scale bars, 50 μm) of the uptake by cultured cortical astrocytes (nuclei stained blue with Hoescht) of 5 kDa FITC-dextran (**j**, green) and 45 kDa TRITC-dextran (**k**, red), respectively, either in the absence of drugs (control, CTRL) or in the presence either of the AQP4 inhibitor TGN-020 (6 µM) or of the A_2A_R antagonist SCH58261 (50 nM) or of both drugs. Quantification of the uptake by cultured astrocytes of the FITC/TRITC-tracers showed that the clearance of these tracers was decreased by the selective AQP4 inhibitor TGN-020, an effect reverted by the selective A_2A_R antagonist, SCH58261 (data are mean ± SEM in the bar graphs, **P < 0.01, *P < 0.05, Student’s *t*-test). **l**, **m** The uptake by cultured astrocytes of the FITC/TRITC-tracers was enhanced by the selective AQP4 activator TGN-073 (10 µM), an effect blunted by the A_2A_R antagonist SCH58261 (50 nM) (data are mean ± SEM, *P < 0.05, one-way ANOVA with Tukey’s multiple comparison test). **n** Representative western blot (out of 3 similar experiments) showing that the pull-down of AQP4 from cultured astrocytes retrieved A_2A_R immunoreactivity (40 kDa and 42 kDa) (third lane from left) compared to non-manipulated membrane extracts (input, first lane from left) and this did not occur with a control IgG antibody (fifth lane from left), indicating that A_2A_R co-immunoprecipitated with AQP4.

Adenosine orchestrates coordinated adaptive responses through adenosine facilitatory A_2A_R and inhibitory A_1_ receptors (A_1_R)^40^. We first observed that 40 Hz light flickering significantly increased A_2A_R density in whole brain extracts (Fig. 7a, b), without altering A_1_R density (Fig. 7a, c). The role of A_2A_R in mediating the increase of glymphatic activity triggered by 40 Hz light flickering was further confirmed using transgenic mice with a deletion of A_2A_R, which did not display an increased CSF influx triggered by 40 Hz light flickering (Fig. 7d–f), as occurred in their WT littermates.

Given the ability of A_2A_R to control CBF^22^, we explored whether 40 Hz light flickering modified CBF in *A*_*2A*_*R*-KO mice. In agreement with our prior findings, 40 Hz light flicking remarkably increased cortical CBF in WT mice (Fig. 7g, h). However, we observed that the increased CBF after 40 Hz light flickering was abolished in *A*_*2A*_*R*-KO mice (Fig. 7g, i).

Apart from controlling CBF, A_2A_R might also control AQP4 as previously suggested^21^ and in alignment with our previous observation that AQP4 activity is crucial for the enhancement of glymphatic flow by 40 Hz light flickering. This adenosine-mediated control of AQP4 was directly confirmed by the direct ability of adenosine to control the activity of AQP4 assessed by measuring the uptake of fluorescein-dextran tracers into cultured cortical astrocytes (Fig. 7j–m). We confirmed that the clearance of these tracers was decreased by the selective AQP4 inhibitor TGN-020 (6 µM), an effect reverted by the selective A_2A_R antagonist, SCH58261 (50 nM) (Fig. 7j, k). Moreover, the direct activation of AQP4 with its selective activator TGN-073 (10 µM) bolstered the uptake of fluorescein-dextran tracers into cultured cortical astrocytes, an effect prevented by the selective A_2A_R antagonist, SCH58261 (50 nM) (Fig. 7l, m). This A_2A_R–AQP4 interplay was further reinforced by the co-immunoprecipitation of AQP4 and A_2A_R from cultured cortical astrocytes (Fig. 7n). This study in cultured astrocytes illustrates the involvement of the A_2A_R-mediated control of AQP4, in parallel to the ability of A_2A_R to control vasomotion.

## Discussion

**Fig. 8:**
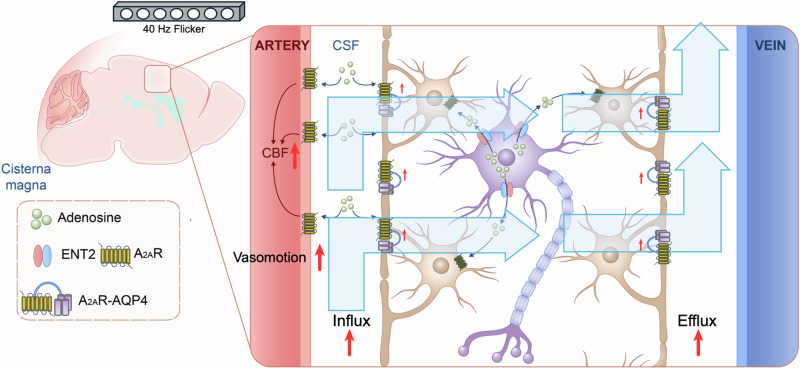
Schematic representation of proposed mechanisms. Light flickering frequency-dependently (specifically at 40 Hz) increases glymphatic influx and efflux. Adenosine-A_2A_R as the signaling pathway linking 40 Hz light flickering with enhanced glymphatic influx through the control of CBF, vasomotion and aquaporin-4: i) 40 Hz flicker enhancement of the glymphatic influx depends on astrocytic AQP4 polarization; ii) genetic deletion of *ENT2* or of *A*_*2A*_*R* abolishes the 40 Hz flicker effects on the glymphatic system; iii) A_2A_R and aquaporin-4 are colocalized and functionally interact in astrocytes.

Gamma sensory stimulation is increasingly recognized as a novel non-invasive strategy to manipulate brain function and afford benefits in different neurodegenerative disorders^12–14^, but its mechanisms of action remain to be determined. On the other hand, the glymphatic system is recognized as fundamental for brain homeostasis since CSF flow in the brain parenchyma eliminates metabolic waste^41,42^. Here, we present direct evidence that 40 Hz light flickering bolstered the activity of the glymphatic system in mice. Specifically, a brief 40 Hz light flickering robustly enhanced glymphatic influx and efflux in a frequency-dependent manner, a process critically dependent on AQP4 activity and involving increased vasomotion. Furthermore, we identified adenosine signaling as a key mediator of the 40 Hz light flickering effect on the glymphatic system by demonstrating its dependence on ENT2-mediated vasomotion and AQP4 polarization and possible interaction between A_2A_R and AQP4 in astrocytes (Fig. 8). The present demonstration that visual stimulation in the gamma frequency bolsters glymphatic flow via adenosine signaling prompts novel pharmacological (targeting ENT2 or A_2A_R) as well as non-invasive (gamma sensory stimulation) strategies for enhancing glymphatic flow to alleviate brain dysfunction.

The first novel conclusion of the present study was the demonstration that 40 Hz light flickering robustly enhanced glymphatic influx and efflux in a frequency-dependent manner in mice. This conclusion was confirmed by the parallel use of three independent methodologies to evaluate glymphatic flow, namely fluorescence tracing, in vivo two-photon microscopy, and DCE-MRI. Furthermore, 40 Hz light flickering-induced facilitation of the glymphatic flow was critically dependent on AQP4, which is essential to sustain glymphatic flow^3,4^, and it was also associated with an increased vasomotion in the brain, which is established to bolster glymphatic flow^30^. Since sleep^18^ and anesthesia^6,27^ are known to markedly improve glymphatic flow, the juxtaposition of our current finding that 40 Hz light flickering bolsters adenosine levels in the brain to increase the glymphatic flow, with the previous evidence that extracellular adenosine builds up as a homeostatic accumulator of the need to sleep^20^ and sleep bolsters the glymphatic system^6^, is suggestive of a role of adenosine in mediating the sleep- and anesthesia-associated bolstering of the glymphatic system. Critically, our findings from two-photon microscopy recording and pre-implanted injection cannula directly confirm that 40 Hz flickering enhances glymphatic flow in awake mice. Thus, 40 Hz flickering improves glymphatic activity in awake animals independently and eventually of top of the impact of sleep.

Our findings also extend the original observation^32^ by demonstrating that flickering visual stimuli drive artery vasomotion and glymphatic flow in the brains, providing hemodynamic mechanisms of the global brain benefits of 40 Hz sensory stimulation apart from the entrainment of gamma activity in particular brain circuits^14,15^. In fact, altering the glymphatic activity impacts on the performance of the whole brain, as heralded by the impact of altered glymphatic flow in mood, memory, and damage in different brain regions caused by ischemia or traumatic brain injury^39,40,42,43^. Thus, this mechanistic relation linking 40 Hz light flickering with the efficiency of the glymphatic system provides a rationale for understanding how 40 Hz light flickering exerts broader effects on processes influencing the whole brain rather than defined circuits.

The second novel conclusion of this study was the critical involvement of ENT2-mediated adenosine signaling in mediating the increase of glymphatic flow induced by 40 Hz light flickering. Thus, the likely driving force for the extracellular adenosine generation by 40 Hz light flickering might be the increased neuronal activity, typified by the entrainment of gamma activity in different brain regions^14,15^, since adenosine is produced in an activity-dependent manner^34,40^. The identification of adenosine as an essential molecular messenger linking 40 Hz light flickering with the enhanced glymphatic influx, was grounded on six parallel observations: (1) 40 Hz light flickering significantly increased adenosine levels in the CSF; (2) the inhibition of the release of adenosine upon inhibition of ENT by dipyridamole treatment, abrogated the ability of 40 Hz light flickering to increase the glymphatic flow; (3) the 40 Hz light flickering enhancement of the glymphatic flow was abolished in *ENT2*-KO mice; (4) 40 Hz flickering increased adenosine levels in the CSF of WT but not *ENT2*-KO mice; (5) genetic deletion of *ENT2* abolished 40 Hz flickering-induced AQP4 polarization; (6) genetic deletion of *ENT2* abolished 40 Hz flickering-induced vasomotion. Altogether, this evidence indicates that 40 Hz light flickering triggers an increased release of adenosine through ENT2 to bolster the activity of the glymphatic system by modulating vasomotion and AQP4 polarization. The ability of 40 Hz light flickering to trigger a robust increase of the extracellular levels of adenosine in the cortex was recently demonstrated by our group^18^, which also corroborated the prime role of ENT2 in agreement with its greater capacity for adenosine transport and its 4-fold higher expression in neurons and astrocytes when compared with ENT1^35,43^.

We further unraveled the role of A_2A_R as the target of the ENT2-mediated release of adenosine responsible for the enhancement of glymphatic flow triggered by 40 Hz light flickering. This was concluded by the abolishment in *A*_*2A*_*R*-KO mice of the enhanced glymphatic flow and CBF-triggered 40 Hz light flickering. We also unveiled that an adenosine-mediated control of AQP4 is involved in the enhancement of glymphatic flow by 40 Hz light flickering. AQP4 has a polarized localization at the paravascular astrocytic endfeet and is crucial for driving the glymphatic flow in the brain’s interstitium and its subsequent clearance from the parenchyma^3,4^. Accordingly, we observed that 40 Hz light flickering failed to augment glymphatic flow in *AQP4*-KO mice. Importantly, 40 Hz light flickering enhanced the polarized expression of AQP4 in astrocytes. However, the interaction between A_2A_R and AQP4 in astrocytes is probably a complex interplay, which encompasses both functional interactions through A_2A_R canonical signaling and possibly direct physically interactions with functional consequences. Functionally, A_2A_R activation can influence AQP4 through classical adenosine signaling pathways, including the modulation of cAMP levels and PKA activity, which may alter AQP4 expression and water channel activity^44^. Physically, A_2A_R and AQP4 may co-localize in astrocytic endfeet and form protein complexes, affecting their respective functions and signaling dynamics. The functional and physical interactions of A_2A_R and AQP4 in astrocytes are in agreement with the previously demonstrated ability of A_2A_R to control AQP4 in an animal model of traumatic brain injury^21^. This tight interplay between A_2A_R and AQP4 reunites in a common framework that previously reported ability of either 40 Hz stimulation^45^ or A_2A_R antagonists^21^ to attenuate functional disability in animal models of brain trauma. These apparent paradoxical effects are attributed to distinct and often opposite effects of the A_2A_R in distinct cellular elements (such as presynaptic vs postsynaptic)^46,47^, different cell types (such as astroglia vs neurons)^48,49^, different brain regions^50,51^ and different pathological stages of the disease^52^. Thus, it is critically important to identify the specific time window where 40 Hz light flickering (and also adenosine-A_2A_R signaling) as well as A_2A_R antagonists exert their distinct therapeutic benefits. In addition, the involvement of A_2A_R in the 40 Hz light flickering-induced increase in glymphatic flow may also result from a combined ability of A_2A_R to increase frequency-dependent neuronal activity^53,54^ and to increase reactive hyperemia^22,55,56^, since neurovascular coupling is increasingly viewed as a driver of the glymphatic system^23,31,32^. Indeed, the 40 Hz light flickering-induced increase in both CBF and glymphatic influx was abrogated in *A*_*2A*_*R*-KO mice. Furthermore, since adenosine also acts as a homeostatic regulator of neurotransmission and synaptic plasticity to restore cellular metabolism in response to stressful conditions^40^, 40 Hz sensory flickering has the additional potential to confer therapeutic benefits by promoting overall brain homeostatic regulation.

Notably, our finding is consistent with recently published independent result^57^, with both studies demonstrating that 40 Hz stimulation enhances glymphatic flow. There are two notable differences between the two studies: (1) Murdock et al. used the combined light and audio stimulation with light intensity at 500 lux, whereas we used 40 Hz light flickering stimulation with light intensity at 3000 lux; (2) while Murdock et al. highlight the crucial role of vasoactive intestinal polypeptide (VIP) neurons in controlling gamma sensory stimulation-induced glymphatic enhancement, we now propose and demonstrate that adenosine signaling may underlie the biological effect of 40 Hz light flickering. Interestingly, a previous study indicated that the tonic activation of A_2A_R is required for VIP signaling in the brain, namely in the hippocampus^58^. Thus, it is possible that adenosine in the cortex may influence the action of VIP cortical neurons to exert its control on the glymphatic system. Future studies will be required to dissect the relation between adenosine and VIP in the control of light flickering-induced increase of the glymphatic activity.

In summary, we conclude that 40 Hz light flickering increased glymphatic flow in awake mice via ENT2-mediated adenosine release and A_2A_R activation, which increased AQP4 polarized expression and vasomotion, to enhance the glymphatic flow. Since deterioration of the glymphatic system occurs in aging^7^ and various neurological disorders contributing to cognitive decline and proteinopathies^42,43,59,60^, the present findings establish 40 Hz light flickering as a novel non-invasive strategy and the adenosine signal as an important modulator to enhance glymphatic flow, with translational potential to therapeutically relieve neurological disorders, with the distinct advantage of promoting sleep, with a remarkable safety profile^15,18^ and with a rapid onset.

## Materials and methods

### Animals

All the experiments were approved and adhered to the guidelines established by the Institutional Ethics Committee for the Use of Animals in Research and Education at Wenzhou Medical University, China, and by the Center for Neuroscience and Cell Biology of the University of Coimbra. Male C57/BL6 mice, aged 9–12 weeks and weighing between 25 and 28 g, were purchased from institute-approved vendors (Beijing Vital River Laboratory Animal Technology Co., Ltd. and the Charles Rivers). *ENT1*-KO mice and *ENT2*-KO mice were generated via CRISPR/Cas9 technology by Beijing Biocytogen and generously furnished by Dr. Yulong Li’s laboratory^29^. *AQP4*-KO mice (Cat# NM-KO-190243) were created by Shanghai Model Organisms Center, Inc. and kindly provided by Dr. Ruiliang Bai’s laboratory. *A*_*2A*_*R*-KO mice and their WT littermates were generated on a C57BL/6 × 129SvEvSteel background through gene targeting procedures as detailed in previously published methods^61^. The mice were housed in a controlled environment with regulated temperature (22.0 ± 2.0 °C) and humidity (60 ± 5%), provided with unrestricted access to both food and water, and adhering to a 12-h light/12-h dark cycle.

### Drugs and fluorescent CSF tracers

Dipyridamole (D9766, Sigma, USA) was solubilized in a 4% methylcellulose solution to achieve a concentration of 1.5 mg/mL. The CSF tracer Y39-1 (FITC-GKTKEGVLYVGSKTK, 2 kDa, NAMICROBIO Co., Ltd, China) was constituted in artificial cerebral spinal fluid (ACSF: 126 mM NaCl, 26 mM HCO_3_, 10 mM glucose, 2.5 mM KCl, 1.25 mM NaH_2_PO_4_, 2 mM MgCl_2_, 2 mM CaCl_2_; pH 7.4) at a concentration of 0.1%. We also designed a fluorescent control peptide Y39-1-mut (FITC-GKTKEGVGFVGTKSK, 2 kDa, NAMICROBIO Co., Ltd, China) by replacing and shifting amino acid fragments, which was handled as Y39-1-mut.

### Intracisternal CSF fluorescence tracer infusion, visualization and calculation

To evaluate glymphatic influx, fluorescent CSF tracers (Y39-1 or Y39-1-mut) were dissolved in ACSF at a concentration of 0.1%. As previously described, mice anesthetized with avertin (Sigma, 120 mg/kg, i.p.) were fixed in a stereotaxic frame with the cisterna magna surgically exposed, and then a 33-gauge needle (the outer diameter of a 33-gauge needle is 0.21 mm and the outer diameter of the needle connection to the tube is 0.47 mm) connected to PE20 tubing (with an inner diameter of 0.38 mm) filled with the tracer was inserted into the cisterna magna (the tube-line system is shown in Supplementary Fig. S5a). 10 µL of the CSF tracer (0.1%) was injected at a rate of 2 µL/min during 5 min with a tubing-nested 10-µL Hamilton syringe (Hamilton, USA). The needle was left in position until completing the brain sampling after the infusion to avoid CSF tracer backflow.

To visualize the movement of the tracer from the cisternal compartments into the brain parenchyma, the animals were sacrificed 30 min after the beginning of the injection and the brain was excised. The brain was fixed by immersion in a 4% paraformaldehyde (PFA) in phosphate-buffered saline (PBS) overnight at 4 °C. Coronal sections (100-μm-thick) were cut with a vibratome (VT1000 S, Leica, Germany) and mounted using Fluoromount G (SouthernBiotech, USA). Tracer influx into the brain was imaged ex vivo by epifluorescence microscopy (DM6B, Leica, Germany) under a 10× objective using six brain sections at 600 μm intervals. Quantitative assessments of the tracer influx were performed by an investigator blind to treatments using ImageJ software. The mean fluorescence intensity and the mean regional fluorescent area (relative to section area) were calculated in the six sections from each animal. Using the ImageJ ROI manager, the brain region of interest was defined as the whole slice. Fluorescent tracer coverage within each region was measured by uniform thresholding and calculating the thresholded area as a percentage of the overall region area, following previously described methods. The fluorescence area coverage and the mean fluorescence intensity from the six brain slices of each animal was averaged to define CSF penetration within a single biological replicate.

### Parenchymal clearance assay

To evaluate the rates of interstitial fluid and solute clearance from the brain, the fluorescent tracer Y39-1 was stereotaxically injected into the brain parenchyma (in the striatum, AP: 0.5 mm, ML: 2.0 mm, DV: –3.25 mm)^9^. In brief, anesthetized animals were fixed in a stereotaxic frame, with the skin gently opened to expose the skull. A total volume of 1 μL Y39-1 (0.1%) was injected at a rate of 0.1 μL/min using a micro-pump (KDS Legato 130, RWD Life Science Co., Ltd., China). The injection syringe was left in position for 5 min, and then withdrew slowly to prevent back-flow. One hour after the intrastriatal injection, the mouse was sacrificed and the brain and deep cervical lymph nodes (dcLNs) were harvested and fixed in PFA overnight at 4 °C. Brain tissues were coronally sliced on a vibratome at 100 μm and mounted. Tracer efflux out of the brain was imaged by epifluorescence microscopy (DM6B, Leica, Germany), with five brain sections, at 600 μm interval, acquired per mouse. The dcLNs were then dehydrated in 30% sucrose solution dissolved in 0.01 M PBS for 2 days and then cut at 40 μm using a cryostat (HM525 NX, ThermoFisher Scientific, USA) for analysis as for brain sections. The processing of images was performed as previously described.

### Light flickering stimulation

The light flickering stimulation procedure followed previous studies^12–14,18^. Mice were placed within a PVC enclosure resembling their home cage but devoid of bedding, and facing two LED bulbs, controlled by a single circuit-relay system in a dark room, situated on opposing sides of the long axis of the enclosure. White LEDs, which emit a spectrum of visible wavelengths spanning 390–700 nm, were employed to administer one of four specific stimulation types: light, 20 Hz, 40 Hz, and 80 Hz flicker, each with 12.5 ms light on and 12.5 ms light off cycles, 60 W and lasting for 30 min. The LEDs were calibrated to have the same duty cycles (50%) and intensities (~3000 lux, ~1.1 mW/cm^2^ at 20 cm distance).

### Ciserna magna cannulation

We used the special copper 29-gauge needles connected to PE10 tubing filled with fresh ACSF for the cisterna magna surgery and MRI acquisition. Copper is a type of diamagnetic metal that exhibits negligible artifacts during MRI scans^62^. The copper used in our study has a very high purity level (over 99.9%), and no artifacts were observed in the captured images. Mice were anesthetized with isoflurane and fixed in a stereotaxic frame. Eye ointment was applied to the eyes. The cisterna magna was exposed and the 29-gauge needle was carefully inserted into the cisterna magna. Then, the needle was fixed in place using dental adhesive and the tubing was sealed with a lighter. After completing cisterna magna cannulation, mice were recovered from anesthesia and stimulated while awake. Subsequently, the CSF tracer was infused into the awake mice, as previously described. Mice were exposed for 30 min to 40 Hz light flickering or normal light, and their brains were harvested 1 h after CSF tracer injection.

### In vivo two-photon fluorescence imaging in awake mice

The CSF tracer influx in the brain of awake mice was visualized through a cranial window using a 2-photon laser scanning microscope (FVMPE-RS, Olympus, Japan)^7^. Three days before microscopy imaging, a unilateral craniotomy (4 mm in diameter) was performed over the prefrontal cortex, 0.5 mm lateral and 1.7 mm anterior to bregma. Great care was taken to prevent dural rupture. Immediately after the craniotomy, the dura was coated with ACSF and sealed with a glass coverslip (3 mm in diameter). Custom-made metal head-fixed plates (Transcend Vivoscope, China) were installed on the mice’s heads. Cisterna imagna cannulation was performed ~2 h prior to imaging. 10 µL of the CSF tracer was infused into the awake mice and mice were exposed for 30 min to 40 Hz light flickering or normal light. To visualize the vascular system, 0.1 mL of blood–brain barrier-impermeable Texas Red dextran 70 (MW 70 kDa; 1% in saline, D1864, Invitrogen, USA) was injected via the caudal vein. The mouse’s head was fixed (Schematic illustration is shown in Supplemental Fig. S5b), and the glass coverslip was positioned under the objective lens. A 25× (1.05 NA) water immersion lens was used to image the cortex, from the surface to a depth of ~120 μm. An Insight X3 laser (Spectra-Physics, Santa Clara, CA) was tuned to 1100 nm for Texas Red and a Mai Tai HPDS-OL laser (Spectra-Physics, Santa Clara, CA) was tuned to 920 nm for FITC. The emission filters were selected as 575–645 nm for Texas Red and 495–540 nm for FITC. Dual-channel acquisition was employed (FITC for tracer movement and Texas Red for the vascular system) with 512 × 512 pixel image acquisition. The cortex was repeatedly scanned from the surface to 120 μm below the surface with 2 μm *z*-steps at 10 min intervals during the duration of the experiment. Image analysis was conducted with ImageJ software. Imaging planes at 80–100 μm below the cortical surface were selected for the analysis of intracisternal tracer penetration.

### Measurement of cerebrovascular vasomotion

Cerebral vascular vasomotion was assessed using in vivo two-photon microscopy, following previously described methods^7^. To measure vessel diameters, 3000 ms X–T line scans were acquired orthogonally to the axis of penetrating arteries located 50–150 μm below the cortical surface. Penetrating arterioles were distinguished from penetrating venules based on morphology: surface arteries passed superficially to surface veins and exhibited less branching at superficial cortical depths. Vessel diameter was determined from X–t plots using ImageJ software, with steady-state diameters calculated as the mean value over the 3000 ms period. Vessel vasomotion (in units of μm*ms) was computed as the absolute area under the diameter–time plot integrated around the running average over the 3000 ms period.

### Polysomnographic recording and analysis

Polysomnographic recording and analysis were performed as previously reported^18^. In brief, four stainless steel screws were implanted to record the EEG, with two located over the primary motor cortex (approximately 2 mm anterior to the bregma and 1 mm lateral to the midline) and the other two over the parietal cortex (approximately 2 mm posterior to the bregma and 1 mm lateral to the midline). Two stainless steel wires were implanted in the neck muscle for electromyogram (EMG) signal tracking. To replicate the experimental conditions of two-photon microscopy, a customized head-fixed titanium head fork was surgically implanted. Subsequently, EEG and EMG electrodes were fixed to a head-mounted device (2631, Bio-Signal Technologies, China), and the entire assembly was secured using dental cement. Following the surgery, mice were returned to their cages. After a minimum of two days for recovery, the mice were immobilized using a two-photon microscopy fixation device. Polysomnographic recordings were simultaneously captured during light treatment initiation. An additional 2-h recording session was conducted upon cessation of the 30-min light treatment to mimic the duration of each two-photon microscopy experiment.

The cortical EEG and EMG signals were amplified, filtered with a high-pass filter above 0.5 Hz and band-pass filtered between 5–45 Hz, digitalized at a 1000 Hz resolution using a tethered data acquisition system (Medusa, Bio-Signal Technologies, China), and synchronized with the infrared video. The sleep stages in the recordings were scored by AI-driven software, Lunion Stage, developed by LunionData (China) (https://www.luniondata.com/en/lunion_stage). The EEG/EMG data collected were analyzed in 4-s epochs and categorized into three stages, namely non-rapid eye movement sleep (NREM), rapid eye movement sleep (REM) and wakefulness (Wake). The scored results were examined and manual adjustments were made as needed. Based on the scored sleep stages, statistical analysis was performed on different vigilance states in the different experimental groups.

### DCE-MRI measurements

MRI was performed using a 9.4 Tesla animal scanner (BioSpec 94/30 USR, Bruker BioSpin, Ettlingen, Germany). A 3-channel phased surface array coil (Bruker) was used as the receiver coil only, and an 86-mm diameter volume coil (Bruker) was used as a transmitter. The surgical procedure to target the cisterna magna was performed as previously described. Catheterized mice were placed on an MR-compatible stereotactic holder with ear bars to restrain the head movement during scanning and were anesthetized maintaining isoflurane induction (0.4%–1%) with a supplement of dexmedetomidine (0.015 mg/kg/h, i.p.) during MRI scanning.

For visualizing the glymphatic flow, DCE-MRI technique employing a 3D T1-weighted FLASH sequence in the sagittal plane was used (TR/TE 14.7/4.6 ms, FA = 15°, Matrix =160 × 128 × 128, FOV = 20 × 16 × 16 mm, averages = 1). The conventional T1-enhancing contrast agent Gd-DTPA tracer was injected into the cisterna magna. The time series T1W scanning protocol was constituted by three baseline scans (12 min) followed by intracisternal infusion of Gd-DTPA (12.5 mM) at a constant rate of 1.0 μL/min for 12 min. Considering the space within the catheter, the actual infused volume of the contrast was 10 μL. Scans continued over 30 measurements spanning 120 min following Gd-DTPA injection.

To overcome potential noise caused by motion during MRI scans, all DCE-MRI data were registered/normalized to baseline DCE-MRI 3D data obtained before Gd-DTPA injection using rigid transformation performed with FLIRT in FSL (http://www.fmrib.ox.ac.uk/fsl)^63^. For B1 correction, we applied semi-quantitative parameters, i.e., the signal changes relative to the baseline level, and we compared the same brain regions between groups. Because we used the same surface coil and tried to place the animal in the same position each time, i.e., the distance between coil and animal brain was kept identical between groups, we believed that the B1 effect will not change significantly of the current results. We also used the infusion rate to describe the contrast agent’s entry into the tissue over time. Infusion rate (IR) is defined as the slope of DCE-MRI time-series signal (S) between the time of arrival (t_a_) and the time at which S reaches its highest value (t_max_) throughout the accumulation phase for each region, which is a semi-quantitative metric describing the influx activity in each brain regions.

$${\rm{IR}}=\frac{S\left({t}_{\max }\right)-S\left({t}_{{\rm{a}}}\right)}{{t}_{\max }-{t}_{{\rm{a}}}}$$

### Immunohistochemistry and quantification of AQP4 polarized localization

Free-floating immunofluorescence staining of 40 μm fixed coronal brain sections was performed to evaluate AQP4 protein localization. Sections were blocked with 10% donkey serum dissolved in PBS with 0.3% Triton X-100 for 1 h at room temperature, followed by incubation with the primary antibody overnight at room temperature. The next day, tissues were rinsed 3 times (10 min/each rinse) in PBS and incubated with the secondary antibody for 4 h at room temperature. The primary antibodies were rabbit anti-AQP4 (1:200; AB3594, Millipore-Merck, USA) and mouse anti-GFAP (1:200; G3893, Sigma-Aldrich, USA). The secondary antibodies were Alexa 555 donkey anti-rabbit (1:500; A-31572, Invitrogen, USA) and Alexa 488 goat anti-mouse (1:500; A-11001, Invitrogen, USA). After washing and mounting, fluorescence was imaged using an epifluorescence microscope (DM6B, Leica, Japan) with a 20× objective, acquiring 40–60 μm *z*-stacks at 2 μm *z*-steps.

AQP4 polarization was evaluated as described previously^7,64^. Image acquisition was performed by an independent investigator with randomized file names to prevent any influence on data interpretation. Image acquisition encompassed capturing equivalent images for the dorsal cortex, lateral cortex, and ventral cortex from each brain section, resulting in three images per section. The mean immunofluorescence intensity was quantified using ImageJ software. For quantification of AQP4 polarization, 50 μm segments centered on blood vessels (as identified by vascular-shaped elements stained with GFAP) were analyzed using the line-plot tool in ImageJ. For polarity calculation, we set the baseline as the average intensity within a 10 μm range, precisely from –20 μm to –10 μm relative to the point of peak fluorescence. The adjusted peak fluorescence values were normalized to the average of the control group value compared in the statistical analysis.

### Western blotting

The brains were extracted and bisected along the sagittal plane, with the cerebellum removed. One-half was utilized for protein extraction and stored at –80 °C. Tissues were lysed by sonication in ice-cold RIPA lysis buffer (P0013B, Beyotime, China) containing phosphatase inhibitors mix (B15001, Bimake, USA) and protease inhibitor cocktail (B14001, Bimake, USA). The protein concentration was determined using the Enhanced BCA Protein Assay Kit (P0010S, Beyotime, China). Subsequently, the samples were diluted in 5× sodium dodecyl sulfate-polyacrylamide gel electrophoresis buffer (P0015, Beyotime, China) and boiled at 95 °C for 8 min. The prepared samples were then loaded onto a 10% polyacrylamide gel for electrophoresis. Gels were transferred to PVDF membranes (Bio-rad). The following primary antibodies were used for western blot analysis: anti-AQP4 (rabbit, 1:1000, AB3594, Millipore-Merck, USA), anti-ENT2 (rabbit, 1:1000, AB181192, Abcam, UK), anti-A_2A_R (mouse, 1:200, SC-365235, Santa Cruz Biotechnology, USA), anti-A_1_R (rabbit, 1:1000, AB3460, Abcam, UK) and anti-β-tubulin (mouse, 1:20,000, 66031-1-Ig, Proteintech, USA). The following secondary antibodies were used: anti-mouse IgG-HRP (goat, 1:3000, GB23301, Servicebio, China) and anti-rabbit IgG-HRP (goat, 1:10,000, BL003A, Biosharp, China).

### Real-time qPCR analysis

The brains were extracted and bisected along the sagittal plane, with the cerebellum removed. One half was utilized for RNA extraction and stored at –80 °C. Then, RNA extraction was performed with the Trizol reagent (Invitrogen, USA). The quantity and quality of the isolated RNA were evaluated using a NanoDrop spectrophotometer. The reverse transcription was conducted using the PrimeScriptTM RT reagent kit (Vazyme, R323, China). The primers used for qRT-PCR included *AQP4* (5′-ATCAGCATCGCTAAGTCCGTC-3′ and 5′-GAGGTGTGACCAGGTAGAGGA-3′), *AQP4*-M1 (5′-CTCCCAGTGTACTGGAGCCCG-3′ and 5′-TGGTGACTCCCAATCCTCCAAC-3′) and *AQP4*-M23 (5′-GGAAGGCTAGGTTGGTGACTTC-3′ and 5′-TGGTGACTCCCAATCCTCCAAC-3′)^65^. Quantitative mRNA analysis was performed using the SYBR Green fluorescence system and a StepOnePlus Real-Time PCR System (Life Technologies, USA), adhering to the manufacturer’s guidelines. Subsequently, mRNA levels were calculated using the ΔΔCt method, with *β-actin* serving as the calibrator gene. The target gene expression was reported as relative expression levels, and each sample underwent analysis in at least triplicates.

### Blood pressure and heart rate measurement

Blood pressure and heart rate were measured using a non-invasive tail-cuff method with a BP-2010A blood pressure device (Softron Biotechnology Ltd., Japan). To mitigate diurnal variations in blood pressure, a consistent time window (9:00–12:00) was selected for measurements of blood pressure in mice. A 3-day acclimatization period was employed, ensuring that mice were accustomed to the procedure. To optimize cardiovascular circulation, mice were enveloped in a cotton sheet and maintained at a controlled temperature of 37 °C within a cylindrical heater, leaving only the tail exposed. A programmable sensor, coupled with an inflatable balloon affixed to the tail-cuff, was used to monitor tail pulse waves and record blood pressure when the pulse waves exhibited stability and rhythmicity. The software read and recorded the blood pressure data. For each mouse, the average value was determined by calculating the mean of five repeated measurements. Blood pressure and heart rate were measured before and after delivering 40 Hz light flickering during 30 min.

### CBF measurement

We measured CBF in the different light-treated groups using Laser Speckle Contrast Imaging/LSCI (RFLSI ZW, RWD Life Science Co., Ltd, China) before and after light treatments. Mice were anesthetized, following which a midline scalp incision was made and retracted to expose the underlying skull. Skull optical clearing was executed as described previously^66^, to enhance the clarity of the cerebrovascular structure. The cerebral cortex was manually outlined, and the mean CBF within the cortical regions of interest (ROIs) was measured.

### UPLC

The mice were randomly divided into 40 Hz flickering group and normal light group. Anesthetized mice were fixed in a stereotaxic frame with the cisterna magna surgically exposed 30 min after stopping light flickering. CSF samples were collected using a pulled glass capillary. Following the puncture, CSF was collected slowly and added to a mixture of precooled inhibitors of adenosine metabolism (20 μL of erythro-9-(2-hydroxy-3-nonyl) adenine hydrochloride (EHNA hydrochloride, a highly potent and selective dual inhibitor of cyclic nucleotide phosphodiesterase 2 (PDE2) and adenosine deaminase (ADA), 20 μg/mL) and 500 μL of dipyridamole (1 mg/mL)) and acetonitrile, following a precise 1:19 (v/v) ratio. The samples were then centrifuged at 13,000 rpm for 10 min. A volume of 100 µL of the supernatant was injected into the UPLC system (Waters, ACQUITY UPLC H-Class Plus, USA) for analysis. Adenosine was separated using an Athena C18 HPLC column (CNW, 2.1 mm × 150 mm, 3 μm particle diameter, Germany) at 25 °C, with a UV detection wavelength of 254 nm. Isocratic elution was performed using a mobile phase consisting of 0.1% formic acid (phase A), and acetonitrile (phase B) at a flow rate of 0.2 mL/min. Standard solutions of adenosine (Sigma-Aldrich, purity > 98%) were prepared over a concentration range from 1 to 1000 ng/mL to construct calibration curves.

### Primary astrocyte cultures

Experiments were performed using astrocytic primary cultures obtained from the cerebral cortex of postnatal Wistar rats for 1–3 days, as previously described^67^. Isolated brain cortices were mechanically and chemically dissociated, using a scalpel and TrypLE reagent (Gibco, Alfagene, Portugal) supplemented with DNAse I (10 mg/mL in 10 mM NaCl, Sigma-Aldrich, USA). Enzymatic digestion was stopped by adding the culture medium: high glucose Dulbecco’s Modified Eagle Medium (DMEM), supplemented with 10% fetal bovine serum (FBS) and 10 mL/L penicillin-streptomycin, pH 7.4. Cell suspension was centrifuged at 115× *g* for 2 min and the pellet was resuspended in culture medium. Cells were then plated in T75 flasks pre-coated with poly-D-lysine (0.1 mg/mL in borate buffer, pH 8.2) at a density of 1 × 10^5^ cells/cm^3^ and maintained in a 5% CO_2_ incubator at 37 °C, until reaching confluence. The culture medium was replaced every 2–3 days. Microglia were removed from the astrocytic monolayer by shaking at 200 rpm, for 2 h at 37 °C, followed by medium replacement to remove microglia in suspension. After the re-establishment of the culture (2–3 h), astrocytes were detached using a mild trypsinization protocol^67^, in which cells were washed with PBS (135 mM NaCl, 2.7 mM KCl, 4.3 mM Na_2_HPO_4_, 1.47 mM KH_2_PO_4_) with 1 mM EDTA and further detached using PBS with 0.05% trypsin (Sigma-Aldrich, USA). Cell suspension was centrifuged at 180× *g* for 5 min and astrocytes were plated in 16 mm or 18 mm coverslips at a density of 3.5 × 10^4^ cells for dextran uptake experiments or 1 × 10^6^ cells in 12-well multiplate for co-immunoprecipitation. Astrocytes remained in culture for 2 days before performing the experiments.

### Dextran uptake permeability assay

Astrocytes seeded in poly-D-lysine-coated coverslips were treated for 24 h with the selective aquaporin-4 inhibitor *N*-1,3,4-thiadiazol-2-yl-3-pyridinecarboxamide (TGN-020, 6 µM, Tocris, UK) or with the selective aquaporin-4 activator N-(3-benzyloxypyridin-2-yl)-benzene-sulfonamide (TGN-073, 10 µM, generously provided by Hironaka Igarashi and Vincent J Huber, Center for Integrated Human Brain Science, Brain Research Institute, Niigata University, Japan) and/or the selective adenosine A_2A_R antagonist 2-(2-furanyl)-7-(2-phenylethyl)-7H-pyrazolo[4,3-e][1,2,4]triazolo[1,5-c]pyrimidin-5-amine (SCH58261, 50 nM, Tocris, UK). The cells were incubated with 3–5 kDa fluorescein isothiocyanate-dextran (8 mg/mL, 30 min, Sigma Aldrich, USA) or 45 kDa tetramethylrhodamine isothiocyanate-dextran (4 mg/mL, 45 min, Sigma-Aldrich, USA) in Krebs buffer (132 mM NaCl, 4 mM KCl, 1.2 mM Na_2_HPO_4_, 1.4 mM MgCl_2_, 6 mM glucose, 10 mM HEPES, 1 mM CaCl_2_, 300 mOsm, pH 7.4), based in a previously described protocol^68^. Cells were then washed 3 times with PBS, fixed with 4% paraformaldehyde for 15 min stained with the nuclei dye Hoescht 333342 in PBS (Sigma-Aldrich, USA) for 3 min. Fixed cells were mounted on microscope slides using fluorescence mounting medium Fluoromount (Sigma-Aldrich, USA) and visualized under an epifluorescence microscope (Axio Imager Z2 microscope with AxioVision Imaging System, version 4.8 from Zeiss, Germany) using a 40× objective. Images obtained from random fields were analyzed using the ImageJ software. Data from fluorescence dextran uptake experiments were imported to ImageJ to manually define ROIs, drawn around each cell body. To define background contribution, ROIs were drawn in regions lacking dye-filled structures and this mean background fluorescence was subtracted to each ROI. On average, 40 cells were analyzed per experiment and the results were expressed as percentages relatively to control (100%).

### Co‑immunoprecipitation

Co-immunoprecipitation was carried out as previously described^69^. Briefly, astrocytes were lysed using RIPA buffer, the lysates were heated for 5 min at 70 °C and DNA was fragmented by passing the sample through a needle. Before the co-immunoprecipitation assay, samples were subjected to a pre-clearing step aiming to reduce non-specific binding and background. For this procedure, lysates were incubated for 30 min at 4 °C under agitation with Protein G PLUS-Agarose (sc-2002, Santa Cruz Biotechnology, USA) and 1 μL of an off-target antibody, anti-IgG (Abcam, the Netherlands). After centrifugation at 100× *g* for 5 min at 4 °C, a sample of the supernatant was collected as input (positive control). The remaining supernatant was processed for immunoprecipitation by incubation with 2.5 μg of anti-AQP4 (Synaptic Systems, Germany) or with the same amount of an irrelevant anti-IgG antibody (for negative control) for 6 h at 4 °C, under agitation. Samples were then incubated with Protein G PLUS-Agarose (Santa Cruz Biotechnology, USA) overnight, at 4 °C, under agitation to pull down the protein complexes. After washing the pellets with RIPA buffer, sample buffer was added and the samples were denatured at 90 °C for 5 min. Beads were removed by centrifugation 1000× *g* for 5 min at 4 °C in filtered Eppendorfs. Samples were analyzed by Western blot^69^ and probed with anti-A_2A_R (1:500, Santa Cruz Biotechnology, USA).

### Statistical analysis

All statistical analysis was performed in Graphpad Prism and Matlab software. Data are presented as the mean ± SEM. *P* < 0.05 was considered to establish statistical significance.

## Supplementary information

Supplemental materials

Direct visualization of 40 Hz light-flickered mice exhibited an earlier rise in signal intensity following Gd-DTPA injection and a greater overall elevation in the brain parenchyma
