# Author Correction: 40 Hz light flickering facilitates the glymphatic flow via adenosine signaling in mice

**DOI:** 10.1038/s41421-025-00845-6

**Published:** 2025-11-18

**Authors:** Xiaoting Sun, Liliana Dias, Chenlei Peng, Ziyi Zhang, Haoting Ge, Zejun Wang, Jiayi Jin, Manli Jia, Tao Xu, Wei Guo, Wu Zheng, Yan He, Youru Wu, Xiaohong Cai, Paula Agostinho, Jia Qu, Rodrigo A. Cunha, Xuzhao Zhou, Ruiliang Bai, Jiang-fan Chen

**Affiliations:** 1 The Eye and Brain Center, State Key Laboratory of Ophthalmology, Optometry and Visual Science, Eye Hospital, Wenzhou Medical University, Wenzhou, Zhejiang China; 2 Oujiang Laboratory (Zhejiang Laboratory for Regenerative Medicine, Vision and Brain Health), School of Ophthalmology & Optometry and Eye Hospital, Wenzhou Medical University, Wenzhou, Zhejiang China; 3 CNC-Center for Neurosciences and Cell Biology, University of Coimbra, Coimbra, Portugal; 4 FMUC-Faculty of Medicine, University of Coimbra, Coimbra, Portugal; 5 Department of Pediatric Sleep, The Second Affiliated Hospital and Yuying Children’s Hospital of Wenzhou Medical University, Wenzhou, Zhejiang China; 6 Key Laboratory of Biomedical Engineering of Education Ministry, College of Biomedical Engineering and Instrument Science, Zhejiang University, Hangzhou, Zhejiang China; 7 Interdisciplinary Institute of Neuroscience and Technology, Zhejiang University School of Medicine, Hangzhou, Zhejiang China; 8 Liangzhu Laboratory, MOE Frontier Science Center for Brain Science and Brain-machine Integration, State Key Laboratory of Brain-machine Intelligence, Zhejiang University, Hangzhou, Zhejiang China; 9 NHC and CAMS Key Laboratory of Medical Neurobiology, Zhejiang University, Hangzhou, Zhejiang China

**Keywords:** Molecular biology, Cell biology

Correction to: *Cell Discovery* 10.1038/s41421-024-00701-z, published online 6 August 2024

In the originally published version of this article, we believed that the notation “These authors contributed equally: Rodrigo A. Cunha, Xuzhao Zhou, Ruiliang Bai, Jiang-fan Chen.” was sufficient to indicate the corresponding author information. Actually, there’s a misunderstanding. One of the co-corresponding authors would not be formally recognized without explicit inclusion of all designated corresponding authors. The correct form should be as follows.

Correspondence: Rodrigo A. Cunha (cunharod@gmail.com) or Xuzhao Zhou (zhouxuzhao@126.com) or Ruiliang Bai (ruiliangbai@zju.edu.cn) or Jiang-fan Chen (chenjf555@gmail.com).

This error does not affect the conclusions of the paper.

