# Supplementary material for: 40 Hz light flickering facilitates the glymphatic flow via adenosine signaling in mice
Source: Cell Discov. 2024 Aug 6;10:81. doi: 10.1038/s41421-024-00701-z (PMC11300858; doi:10.1038/s41421-024-00701-z)
Supplement: Supplementary file 1 — Supplemental materials [file 41421_2024_701_MOESM1_ESM.docx]

**Supplemental information titles and legends**

**Supplemental Video S1.** **Direct visualization of 40 Hz light-flickered mice exhibited an earlier rise in signal intensity following Gd-DTPA injection and a greater overall elevation in the brain parenchyma.**

**
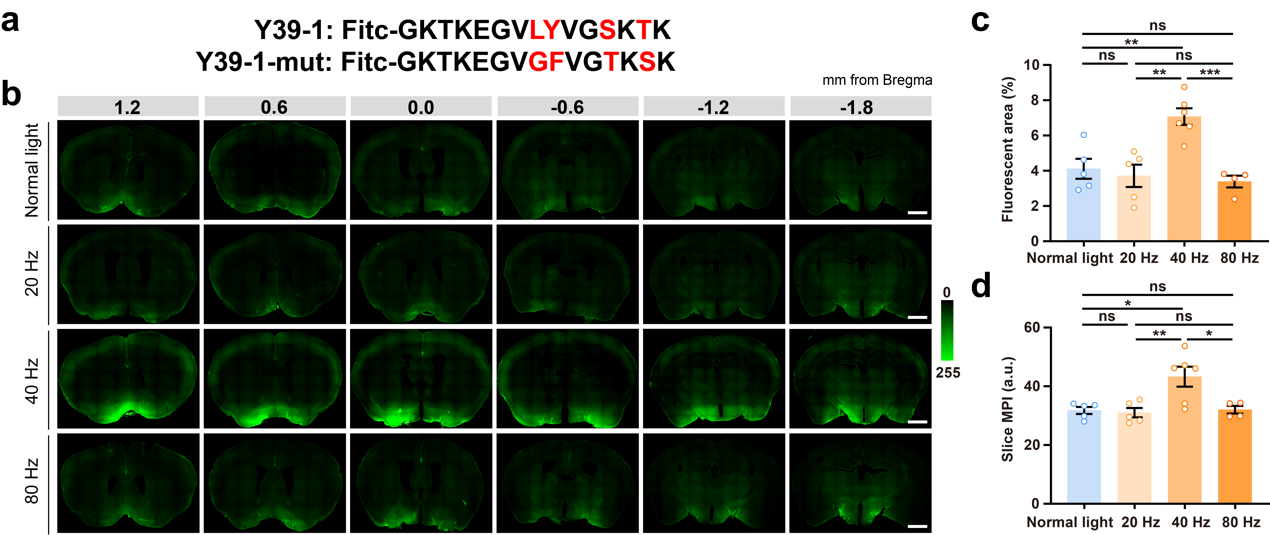
**

**Supplemental Fig. S1. 40 Hz light flickering enhanced glymphatic influx measured by Y39-1-mut tracer. a** Amino acid sequence of Y39-1 and Y39-1-mut fluorescent peptides. **b** Representative images showing that 40 Hz light flickering increased the parenchymal distribution of Y39-1-mut at 30 min after intracisternal injection; numbers indicate the anteroposterior distance from bregma in mm (scale bar, 1 mm). **c, d** Quantification of intracisternally injected Y39-1-mut mean pixel intensity (MPI, in arbitrary units, a.u.) and fluorescent area (expressed as % of section area) in whole sections 30 min after exposure to 20 Hz, 40 Hz or 80 Hz light flickering; analysis was performed on six sections per animal (n = 4-6 mice/group, mean ± SEM in the bar graphs, ***p < 0.001, **p < 0.01, *p < 0.05, ns - not significant, one-way ANOVA with Tukey’s multiple comparison test).

**
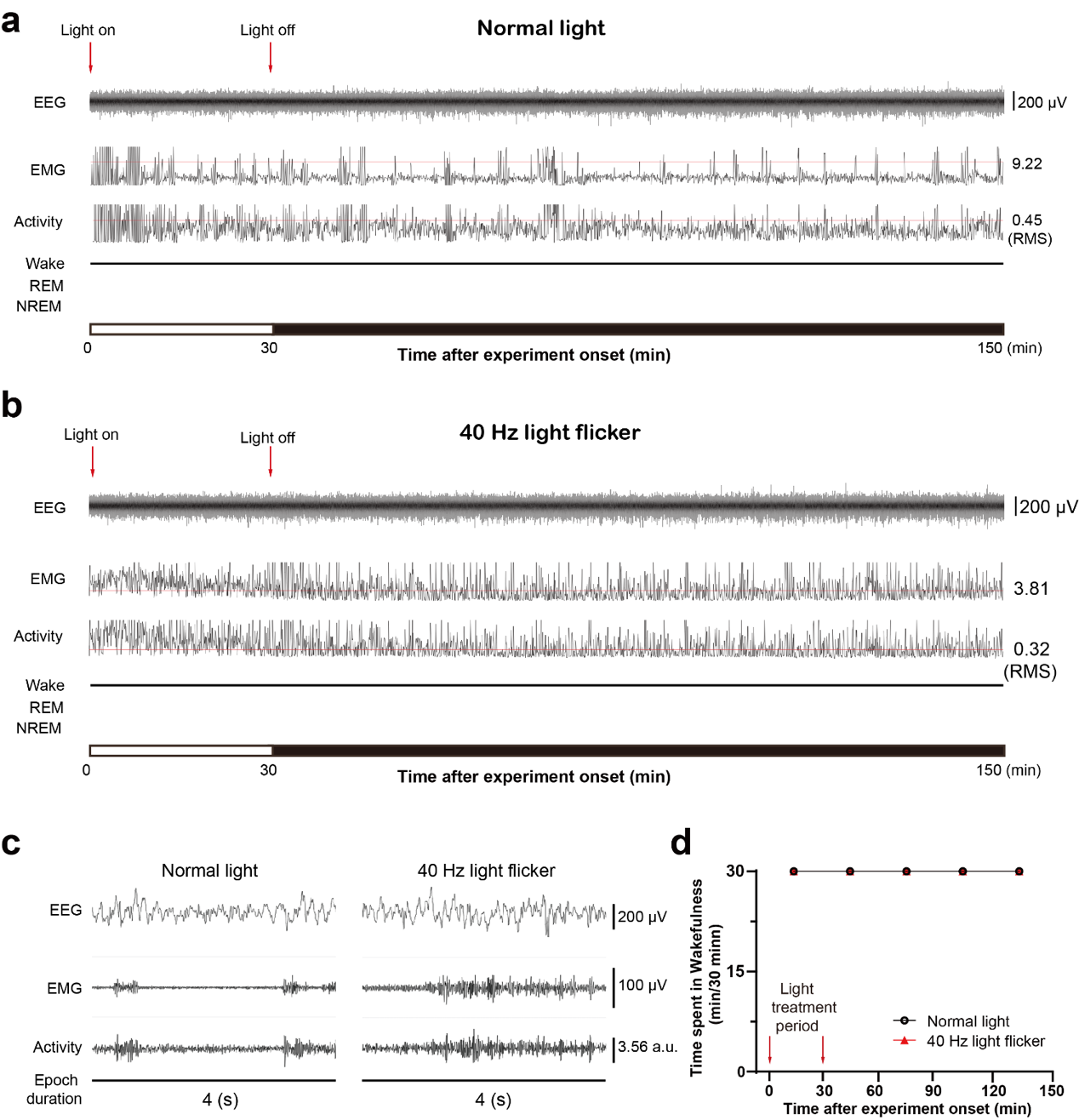
**

**Supplemental Fig. S2. Mice exhibited wakefulness during two-photon microscopy experiments. a, b** Representative EEG, EMG, activity profiles and representative curve of vigilance stages’ changes in WT mice after the normal light or 40 Hz light flicker treatment under the same experimental conditions as in the two-photon microscopy experiment. **c** Representative EEG, EMG, activity profiles of a 4-second analysis epoch in mice after normal light and 40 Hz light flicker treatment, respectively. **d** Time-course of wakefulness in mice after normal light and 40 Hz light flicker treatments under the same experimental conditions as in the two-photon microscopy experiment.

**
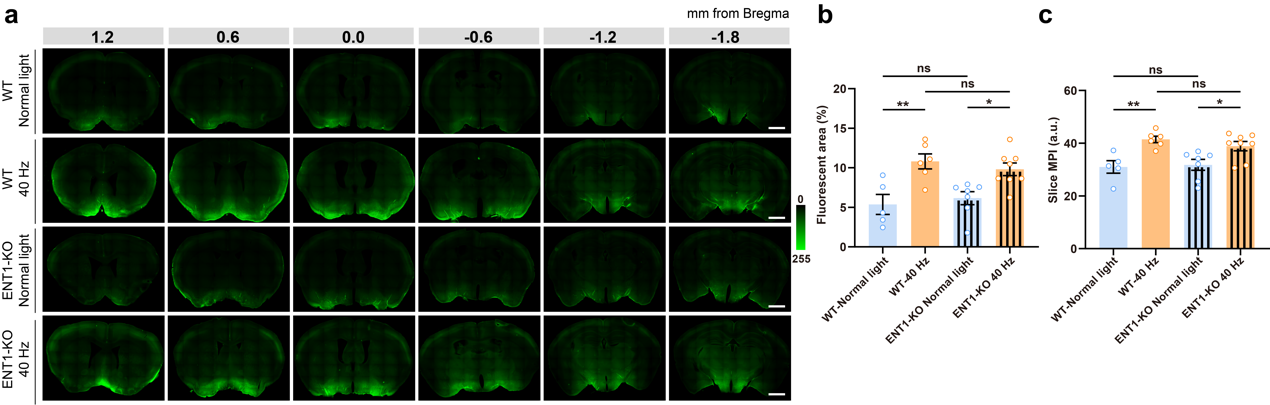
**

**Supplemental Fig. S3. 40 Hz flickering still significantly increased glymphatic flow in ENT1-KO mice. a** Representative photographs of fluorescence in coronal brain section collected 30 min after injection of the Y39-1 fluorescent tracer in the cisterna magna of wild type mice (top two rows) or ENT1 knockout mice (bottom two rows), previously exposed either to normal light or to 40 Hz light flickering during 30 min; numbers indicate the anteroposterior distance from bregma in mm (scale bar, 1 mm). **b, c** Quantification of intracisternally injected Y39-1 mean pixel intensity (MPI, in arbitrary units, a.u.) and of the fluorescent area (expressed as % of section area), showed that exposure to 40 Hz light flickering increased the glymphatic influx in wild type (WT) mice and ENT1 knockout (KO) mice; analysis was performed on six sections per animal (n = 5-8 mice/group, mean ± SEM in the bar graphs, *p < 0.05, **p < 0.01, ns - not significant, one-way ANOVA with Tukey’s multiple comparison test).


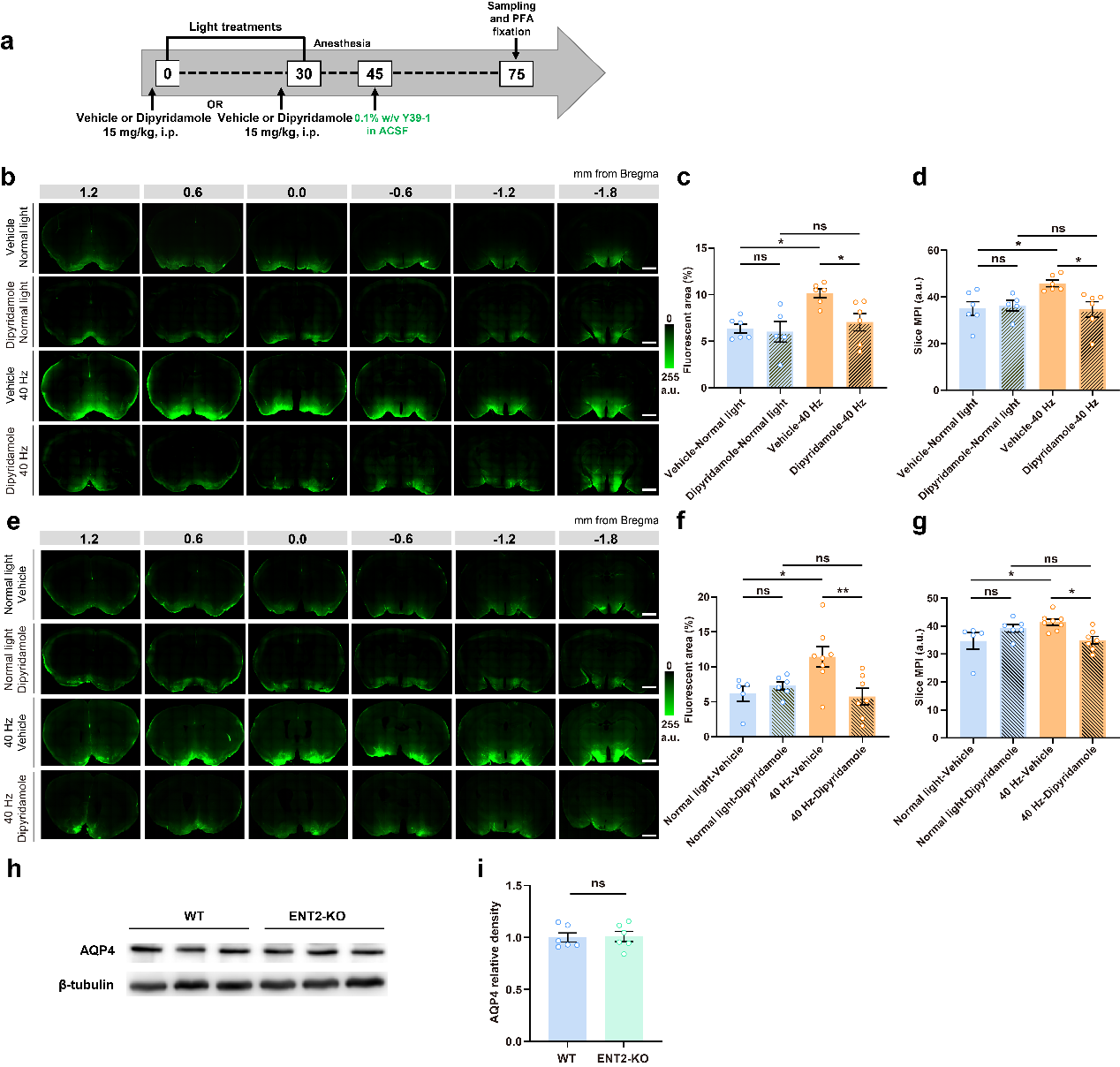


**Supplemental Fig. S4.** **The ability of 40 Hz flickering to increased glymphatic flow is abolished by the pharmacological inhibition of ENTs with dipyridamole. a** Schematic protocol showing that the ENT1/2 inhibitor dipyridamole (15 mg/kg) was administered intraperitoneally either at the time of initiation of light flickering exposure or near the end of light stimuli. **b** Representative images showing that administering dipyridamole at the time of light flickering blunted the ability of 40 Hz light flickering to modify Y39-1 fluorescence in the brain parenchyma; numbers indicate the anteroposterior distance from bregma in mm (scale bar, 1 mm). **c**-**d** Quantification of intracisternally injected Y39-1 mean pixel intensity (MPI, in arbitrary units, a.u.) and of the fluorescent area (expressed as % of section area) in whole sections; analysis was performed on six sections per animal (n = 5-6 mice/group, mean ± SEM in the bar graphs, *p < 0.05, ns - not significant, one-way ANOVA with Tukey’s multiple comparison test). **e** Representative images showing that administering dipyridamole near the end of light stimuli blunted the ability of 40 Hz light flickering to modify Y39-1 fluorescence in the brain parenchyma; numbers indicate the anteroposterior distance from bregma in mm (scale bar, 1 mm). **f, g** Quantification of intracisternally injected Y39-1 mean pixel intensity (MPI, in arbitrary units, a.u.) and of the fluorescent area (expressed as % of section area) in whole sections; analysis was performed on six sections per animal (n = 5-8 mice/group, mean ± SEM in the bar graphs, *p < 0.05, **p < 0.01, ns - not significant, one-way ANOVA with Tukey’s multiple comparison test) **h** Representative Western blot (out of 3 similar experiments) and quantification **i** of AQP4 and β-tubulin densities in whole brain extracts of wild type (WT) mice and ENT2 knockout (KO) mice (n = 6 mice/group, mean SEM in bar graphs, ns - not significant, unpaired student’s t test).


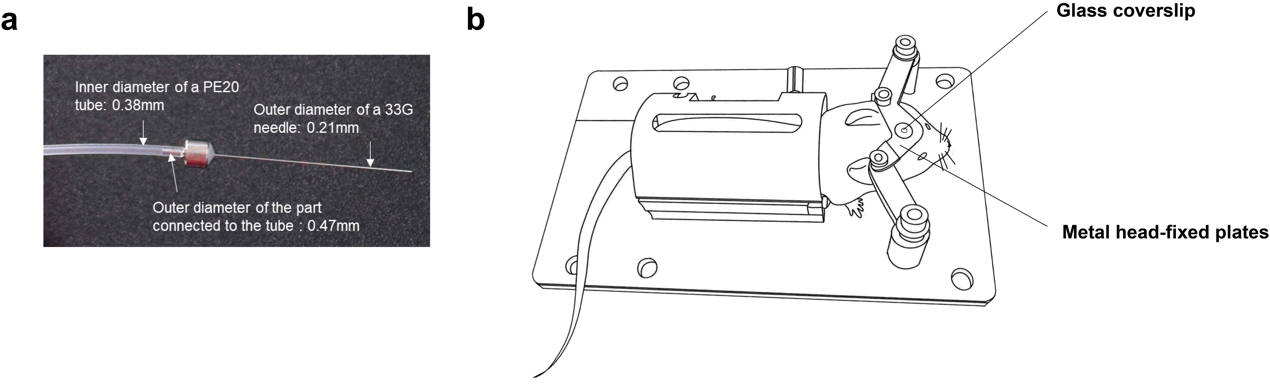


**Supplemental Fig. S5.** **Experimental schematic illustration. a** Schematic illustration of cisterna magna injection needle. **b** Schematic illustration of two-photon fluorescence imaging in awake mice.
